# Identification of Rice Seed-Derived *Fusarium* spp. and Development of LAMP Assay against *Fusarium fujikuroi*

**DOI:** 10.3390/pathogens10010001

**Published:** 2020-12-22

**Authors:** Hubiao Jiang, Na Wu, Shaomin Jin, Temoor Ahmed, Hui Wang, Bin Li, Xiaobi Wu, Yidan Bao, Fei Liu, Jing-Ze Zhang

**Affiliations:** 1 State Key Laboratory of Rice Biology and Ministry of Agriculture Key Lab of Molecular Biology of Crop Pathogens and Insects, Institute of Biotechnology, College of Agricultural and Biotechnology, Zhejiang University, Hangzhou 310058, China; 371112@zju.edu.cn (H.J.); 119287@zju.edu.cn (S.J.); temoorahmed@zju.edu.cn (T.A.); 21916180@zju.edu.cn (H.W.); libin0571@zju.edu.cn (B.L.); 2 College of Biosystems Engineering and Food Science, Zhejiang University, 866 Yuhangtang Road, Hangzhou 310058, China; nawu018@zju.edu.cn (N.W.); ydbao@zju.edu.cn (Y.B.); fliu@zju.edu.cn (F.L.); 3 Agricultural and Rural Bureau of Cangnan County, Wenzhou 325000, China; wxb13758857075@gmail.com

**Keywords:** bakanae, rice seed, fungal taxonomy, phylogeny, LAMP, *Fusarium fujikuroi*, diagnostics

## Abstract

*Fusarium* species are important seedborne pathogens that cause rice bakanae disease (RBD). In this study, 421 strains were isolated from 25 rice samples collected from Zhejiang, Anhui, and Jiangxi provinces of China. Furthermore, 407 isolates were identified as *F. fujikuroi* (80.05% isolation frequency), *F. proliferatum* (8.31%), *F. equiseti* (5.94%), *F. incarnatum* (2.61%), *F. andiyazi* (0.95%), and *F. asiaticum* (0.48%) based on morphology and translation elongation factor 1-alpha (TEF1-α) gene. Phylogenetic analysis of combined sequences of the RNA polymerase II largest subunit (RPB1), RNA polymerase II second largest subunit (RPB2), TEF1-α gene, and ribosomal DNA (rDNA) internal transcribed spacer (ITS) showed that 17 representative strains were attributed to six species. Pathogenicity tests showed that representative isolates possessed varying ability to cause symptoms of bakanae on rice seedlings. Moreover, the seed germination assay revealed that six isolates had different effects, such as inhibition of seed germination, as well as seed and bud rot. The loop mediated isothermal amplification (LAMP)-based assay were developed for the detection of *F. fujikuroi.* According to sequences of desaturase-coding gene promoter, a species-specific marker desM231 was developed for the detection of *F. fujikuroi.* The LAMP assay using seeds collected from field was validated, and diagnostics developed are efficient, rapid, and sensitive.

## 1. Introduction

Rice (*Oryza sativa* L.) is one of the most important staple foods that feeds more than 60% of the world’s population [1]. Rice bakanae disease (RBD) caused by seed-borne *Fusarium spp*. may lead to a global food crisis. The disease symptoms of RBD are slender, chlorotic, and elongated primary leaves [2], due to the production of fungal gibberellic acid, which has resulted in the disease being called the Japanese word ‘bakanae’, which means the ‘foolish seedling’ [3]. The disease incidence usually ranges from 0.25% to 20% [4], even up to 40% in India [5], leading to a severe rice yield loss [6]. The pathogens responsible for the disease are primarily seedborne, but it also survives in soil and infected plant debris, which persists from one season to the next from the remains of infected plants [2]. Although species of the *F. fujikuroi* species complex (FFSC or *fujikuroi*) [7], such as *F. andiyazi*, *F. fujikuroi*, *F. proliferatum*, and *F. verticillioides*, are associated with bakanae of rice, *F. fujikuroi* is believed to be the core species responsible for the symptoms and was detected only in seed samples from Asia [8]. Besides, other species, like *F. equiseti*, *F. graminearum*, *F. oxysporum*, *F. avenaceum*, *F. brevicatenulatum*, *F. begonia*, *F. sterilihyphosum***,** *F. napiforme*, and *F. sporotrichioides***,** occasionally, also were isolated from seeds [4,9,10]. In China, bakanae disease caused by *F. fujikuroi* has been reported [8,11]; however, pathogens and their virulence on rice seeds have still not been investigated.

*Fusarium* spp. associated with RBD can reduce seed germination and possessed varying ability to cause symptoms of bakanae on rice, while *F. fujikuroi* had the stronger pathogenicity than others in the seedlings [8], being involving in gibberellin A3 production. FFSC produce a wide range of mycotoxins, including fumonisins (FBs), moniliformin (MON), beauvericin (BEA), fusaproliferin (FUP), enniatins (ENNs), and fusaric acid (FA) [11], while, in individual species, this ability seems to be species-specific, e.g., fumonisins are easily produced by members of *F. proliferatum* and *F. verticillioides* but trace fumonisins in *F. fujikuroi* and *F. andiyazi* [12]. All species produce BEA, and other species produce MON and ENNs, except without ENNs product in *F. verticillioides* but *F. begoniae* and *F. verticillioides* and MON in *F. sterilihyphosum* and *F. begoniae* [13]. In contrast, gibberellins GA3 production (from its precursors GA4 and GA7) was found only for the species *F. fujikuroi* [8,13]. However, interestingly, analysis of gibberellins biosynthetic genes showed that isolates of *F. proliferatum* and *F. fujikuroi* contained entire gibberellins gene cluster except *F. verticillioides* with only two genes (desaturase and P450-4) [14]. The reason for no gibberellin production in other species was considered to be associated with a gene-related mutation [15]. Gibberellins biosynthesis pathway in the FFSC has been well documented [16,17,18]. In this pathway, the desaturase (Des) plays an important role in conversion of GA14 to GA7, and, subsequently, GA7 is converted to the main product in *F. fujikuroi*, GA3, by 13-hydroxylation (P450-3) (Supplementary Figure S5) [16]. Our results also found a more considerable sequence difference of the Des-coding gene promoter in other *Fusarium* spp., comparing with *F. fujikuroi,* possibly involved in the loss of promoter function (Supplementary sequence 2). Recently, various species complexes found on rice seeds include *F. sambucinum* species complex (FSSC or *sambucinum*) [7,19], *F. incarnatum-equiseti* species complex (FIESC or *incarnatum-equiseti*) [20], and *F. chlamydosporum* species complex (FCSC or *chlamydosporum*) [7,10]. However, in China, diversity and toxigenic potential of *Fusarium* spp., associated with rice bakanae, are lacking.

Some species in the FFSC surviving in rice seeds are transmitted through long-distance seed transport. After sowing, pathogens develop and infect the young seedlings to cause the disease symptoms. In order to prevent pathogen, spread, and disease occurrence, early detection of pathogens in seeds and seedlings is necessary. Molecular techniques based on DNA sequences have been extensively used to identify fungal species, such as polymerase chain reaction (PCR) using species-specific primers, and relative quantification real time PCR have verified to be powerful tools for detection and identification [21,22]. However, due to the need for specialized equipment, these techniques cannot be used in the field [6]. In control, loop mediated isothermal amplification of DNA (LAMP) with an inexpensive device (single temperature) and extremely high amplification efficacy shows significant advantages over PCR and has been used for the detection of plant pathogens under complex conditions. In the detection of pathogens associated with RBD, although a few markers, such as TEF1-α gene [23], intergenic spacer (IGS) region [24], and non-ribosomal peptide synthetase gene (NRPS31) [8,25], were used to develop PCR- or LAMP-based detection for *F. fujikuroi*, gibberellins GA3 metabolite production-related specific markers for the identification of *F. fujikuroi* is not reported.

The Anhui, Zhejiang, and Jiangxi provinces are the important rice-growing areas in China (Supplementary Figure S1), and the bakanae disease usually occurs seriously in fields (Supplementary Figure S2), but *Fusarium* spp. associated with the disease on rice seeds and their virulence have not been documented. In this study, therefore, isolates of *Fusarium* species on rice seeds were isolated and identified by combination of morphological and molecular data, their pathogenicity was determined, and a sequence specific marker was developed for detection of dominant pathogen in rice seeds.

## 2. Materials and Methods

### 2.1. Seed Samples, Fungal Isolation, and Preliminary Identification

The rice seed samples, which were widely cultivated cultivars were collected from Zhejiang, Anhui, and Jiangxi provinces. A total of 25 seed samples, representing 23 varieties that were widely grown locally, were obtained (Supplementary Table S1). Rice seed samples for each variety was collected by a seed sampler using five bags (25 kg). After being mixed, one-hundred seeds of each sample were randomly selected [26], and they were placed into five plates (20 seed per plate) containing peptone-Pentachloronitrobenzene (PCNB) agar (PPA) medium (Peptone 15 g, KH_2_PO_4_ 1.0 g, MgSO_4_•7H_2_O 0.5 g, PCNB (Pentachloronitrobenzene) 750 mg, Agar 20 g, H_2_O to 1 L), which was a selective isolation medium for *Fusarium* spp. [27]; the plates were incubated at 25 °C for seven days. One isolate from per seed were selected, and it was moved into a plate containing potato dextrose agar (PDA, Guanzhi Biological Engineering Co. LTD, Shanghai, China) under ultraviolet (NUV) light for the single-spore isolation. The single-spore isolates were preliminarily identified based morphological characteristics on carnation leaf agar [28] and combination of sequencing of translation elongation factor 1α (TEF1-α) gene [9], as described below. The sequences of TEF1-α gene were analyzed by using the NCBI Basic Local Alignment Search Tool (BLAST) program in GenBank database.

### 2.2. Morphological Identification

To observe the morphological characteristics, the isolates were cultured on carnation leaf agar (CLA; 20 g/L bacto-agar, 5~10 carnation leaf pieces) [28] and PDA. The carnation leaf pieces were prepared from fresh carnation leaves. The leaves were cut into about 5 mm^2^ pieces, dried in the air oven at 70 °C for 3~4 h, and then were sterilized using gamma irradiation from a Cobalt 60 source (Zhejiang University Testing Center, Hangzhou, Zhejiang, China). Fourteen days after culturing at 25 °C with NUV (near ultra violet), fungal asexual structures (the shape and size of microconidia, macroconidia, and conidiophores, length of microconidia chains, and sporodochia formation on carnation leaf pieces) were observed and measured using a Zeiss Axioskopt2 microscopy (Carl Zeiss Microscopy Company, Germany) with Axiocam CCD (charge coupled device) camera and Axiovision digital imaging software (AxioVision Software Release 3.1., v.3–2002; Carl Zeiss Vision Imaging Systems)(Carl Zeiss Microimaging Company, Jena, Thuringen, Germany).

### 2.3. DNA Extraction, PCR Amplification, and Sequencing

All *Fusarium* isolates were cultured on potato dextrose broth (PDB) at 25 °C for five days. The fungal mycelia were harvested and grounded to a fine powder in liquid nitrogen. Genomic DNA was extracted by using a rapid fungal genomic DNA extraction kit (Sangon, Shanghai, China) according to the manufacturer’s protocols. The genomic DNA obtained was resuspended in 50 μL Tris EDTA (ethylene diamine tetraacetic acid, TE) buffer and stored at −20 °C after the concentration of genomic DNA in each sample was quantified determined by a NanoDrop 2000 spectrophotometer (Thermo Scientific Inc., Wilmington, DE, USA).

To amplify sequences of different loci, the primer pairs EF1/EF2 for TEF1-α gene were used for PCR amplification of all isolates [29]. The representative isolates were selected for other PCR amplification according to geographical location, cultivation area of varieties and isolation frequency. The primer pairs RPB1-Fa/RPB1-G2R were used for PCR amplification for the RNA polymerase largest subunit gene (RPB1) [30], RPB2-5f2/RPB2-7cr for the RNA polymerase second largest subunit gene (RPB2) [30], and ITS (internal transcribed spacer) 6/ITS4 for amplification of ribosomal DNA (rDNA) ITS region. The PCR amplification was performed in a 25 μL reaction volume with 12.5 μL 2 × Taq Master (Sangon, Shanghai, China), 1.0 μL of each primer (10 μM), and 1 μL template DNA (2 μg/μL). The thermal cycling program was performed with 30 cycles after an initial denaturation at 95 °C for 3 min. Each cycle included a denaturation step at 95 °C for 1 min, annealing at a suitable temperature for 1 min, and an extension step at 72 °C for 2 min. Annealing temperatures for each reaction were 53 °C for ITS region, 55 °C for RPB1, RPB2, and 54 °C for TEF1-α. The amplified products were sent to the Hangzhou TsingKe Science and Technology Co., LTD (Hangzhou city, China), for sequencing in both directions.

### 2.4. Phylogenetic Analysis

The obtained sequences were analyzed by using BioEdit software [31], and they were deposited in the GenBank database (Table 1). Additional sequences mainly representing cultures from the *Fusarium* MLST (multilocus sequence typing) database (http://www.westerdijkinstitute.nl /fusarium/) and the NCBI’s (National Center for Biotechnology Information) GenBank were downloaded and relevant sequences were included in the subsequent phylogenetic inference (Table 1). The sequences of each gene were aligned with MAFFT (multiple alignment using fast Fourier transform, Kyoto University, Japan) 7.273 [32] and trimmed with BioEdit. The resulted alignment was put into the Gblocks 0.91 b to eliminate the ambiguously aligned positions and divergent regions prior to phylogenetic analyses [33].

The phylogenetic trees were constructed with maximum likelihood (ML) and Bayesian inference (BI) methods. The model of evolution applied to each alignment was estimated using jModel Test 2.1.7 [34] and the model chosen according to the Akaike information criterion. The best TIM1ef + I + G model was selected for RPB1, and CTR + I + G, TIM2 + G and JC + C for RPB1, TEF1-α and ITS, respectively. ML analyses were performed on the concatenated different sequences to produced different data sets, including RPB1 + RPB2 for genus *Fusarium* spp., RPB1+ RPB2 +TEF1-α for the F*SS*C (s*ambucinum*) and FFSC (*fujikuroi*), and RPB2 + ITS + TEF1-α for FIESC (*incarnatum-equiseti*), with RaxmlGUI v. 1.5 [35]. ML bootstrap (ML-BS) analysis for each ML tree was performed with 1000 fast bootstrap replicates with the same parameter settings using the different model of nucleotide substitution for different data sets. A threshold of ≥80% was used as the cut-off for significantly supported nodes.

BI analyses were conducted with MrBayes v. 3.2.6 run by partitioning codon positions [36]. The best TrNef + I + G model was selected for RPB1 and RPB2, and TIM2ef + G and JC for TEF1-α and ITS. The Markov-chain Monte Carlo searches were performed with four chains, each of which was run for 10,000,000 generations, with trees sampled every 100 generations. The initial 25% of trees from each run were discarded as burn-in, and the remaining trees were combined into one tree with 50% majority rule consensus tree. BI posterior probability (BI-PP) values equal or above 0.95 were found to be significant. The resulted trees were imported into FigTree v.1.3.1. (http://tree.bio.ed.ac.uk/software/figtree/).

### 2.5. Pathogenicity Tests

The pathogenicity tests were performed to confirm the changes in virulence among different *Fusarium* species by using inoculated seeds of the rice cultivar (*Oryza sativa* L., cv. Jinzao 47). The rice seeds were sterilized by soaking in 1% sodium hypochlorite solution for 10 min, washed three times with sterile water, and then left to dry on sterile filter paper in a flow cabinet. For inoculum preparation, each single spore isolate, including *F. fujikuroi* ZJ01, *F. andiyazi* ZJ08, *F. proliferatum* ZJ05, *F. incarnatum* ZJ11, *F. asiaticum* ZJ10, and *F. equiseti* ZJ09, was cultured on PDA for 10 days; each plate was flooded with sterile distilled water, and spores were harvested by filtration with sterile miracloth. The spore suspensions were prepared and adjusted to 1 × 10^6^ spores/mL using a hemocytometer, as described by Choi et al. [9]. The sterile seeds were soaked in the spore suspensions for 36 h or in sterile water as control. The eight inoculated seeds were sowed into a pot containing an autoclaved mixture of soil and sand in the ratio of 3:1. Each treatment had three replications for each isolate. The pots were placed in a greenhouse in which temperature was maintained at 28 °C during the day and 25 °C during the night. The whole test was repeated twice. The seedlings were observed daily after symptoms appeared.

The incidence of disease symptoms could start from the stage of seed germination due to the host-pathogen interactions. The seed germination assays were used to confirmed the effect six *Fusarium* species on seed germination. The sterilized seeds were placed into filter papers in a plate, 50 μL spore suspension (1 × 10^6^ spores/mL) prepared above was added to each seed, and the seeds were dried in a laminar flow cabinet. The 20 inoculated seeds were transferred to a plastic tissue culture vessel (480 mL) containing three layers of wet filter paper, and the vessels were placed in incubators under a 12:12 h, light: dark, 28:25 °C regime. The control seeds were treated with sterilized water, and each treatment (20 seeds) was repeated five times for each isolate, and whole assay were repeated twice. The disease symptoms were observed daily and the percentage inhibition (PI) of seed germination was calculated using the formula PI (%) = [(C − N) /C)] × 100, where C was number of seed germination in control, and N was number of seed germination in different treatments by inoculation with fusarium isolates.

### 2.6. LAMP Primer Design

The dominant species associated with bakanae disease were selected as the target for detection based on identification results on rice seeds. If the dominant species was *F*. *fujikuroi*, sequences of enzyme-coding genes and their promoters in gibberellins biosynthesis pathway [17] were analyzed using genome data of *Fusarium* spp. in the GenBank database. If the conjectural target sequences were found, they were analyzed after PCR amplification and sequencing using the identified isolates. Once sequences of species-specific markers were obtained, LAMP primer design would be conducted using LAMP Primer Explorer V5 software (http://primerexplorer.jp/e/).

### 2.7. LAMP Reaction and Optimization of Its Condition

The LAMP reaction was performed in a 25 μL reaction mixture containing 3.0 μL 10 × Thermo Pol Buffer (200 mM Tris-HCl pH 8.8, 100 mM KCl, 100 mM (NH_4_)_2_SO_4_, 20 mM MgSO_4_, 1% Triton X-100) (Sangon, Shanghai, China), 4.5 μL MgSO_4_ (100 mM), 3.5 μL dNTPs (10 mM), 4 μL betaine (5 M) (Sangon, Shanghai, China), 2 μL each of FIP and BIP (40 mM), 0.5 μL each of F3 and B3 (10 mM), 1 μL each of LF and LB (10 mM), 2.0 μL template DNA, and 1 μL Bst DNA Polymerase Large Fragment (8 U/μL; New England Biolabs, UK). The reaction mixture was incubated at 50, 55, 60, 65, 67, 68, 69, and 70 °C, for 55 min to determine the optimal conditions for LAMP assay. For optimal time, the reaction mixture was incubated at 65 °C for 20, 25, 30, 35, 40, 45, 50, 55, 60, 65, 70, and 75 min, respectively. Each reaction was terminated at 80 °C for 10 min. Each sample was amplified for LAMP assays with three independent replicates. The LAMP products were visualized by color change after adding 0.25 μL 10000× SYBR Green I (Sangon, Shanghai, China) and a short vortex or by 1% agarose gel electrophoresis.

### 2.8. Specificity and Sensitivity of LAMP Reaction

For testing stability of LAMP primers, 15 isolates of *F*. *fujikuroi* from different provinces were used for the detection of LAMP amplification. A total of 14 isolates of *Fusarium*. spp. and nine non-*Fusarium* species were used in this study to determine the specificity of LAMP primers (Supplementary Table S2). The genomic DNA for each isolate was extracted, as described above. The LAMP amplification for isolate were conducted at 65 °C for 60 min. The LAMP products were observed by a color change and product electrophoresis as described above. The PCR amplification of isolate were performed using the F3/B3 primer pair in an automated thermal cycler (Eppendorf AG, Germany), mainly including 54 °C annealing temperature and 30 cycles of denaturation in 25 μL reaction volume to confirm the reliability as described by Li [37]. The purified PCR products were verified by 1.0% agarose gel electrophoresis. The genomic DNA from *F*. *fujikuroi* was serially diluted from 9.4 ng/μL to 94 ag/μL to determine the LAMP assay’s sensitivity. The LAMP amplification was performed as described above. PCR amplification was used as a control. The LAMP and PCR amplification for each concentration were repeated three times.

### 2.9. Detection of Rice Seed Samples

In order to determine the applicability of the LAMP assay on rice seeds, 30 seed samples were obtained. The rice seed samples were collected from infected (with typic symptoms of bakanae disease) or healthy rice panicles in 18 parcels of rice field with cv. Jinzao 47 during the harvest period in Fuyang city, Zhejiang province, and dried as general rice harvest procedure. After storage for 30 days, the genomic DNA of seeds was extracted using the Plant Direct PCR Kit (TsingKe Xinye biotechnology co., LTD, Beijing, China) according to the manufacturer’s protocol. The LAMP amplification was performed as described above, and PCR amplification was used as control. LAMP and PCR amplification for each sample were repeated two times.

## 3. Results

### 3.1. Isolation and Identification Using Morphology and TEF1-α Gene Sequences

**Figure 1:**
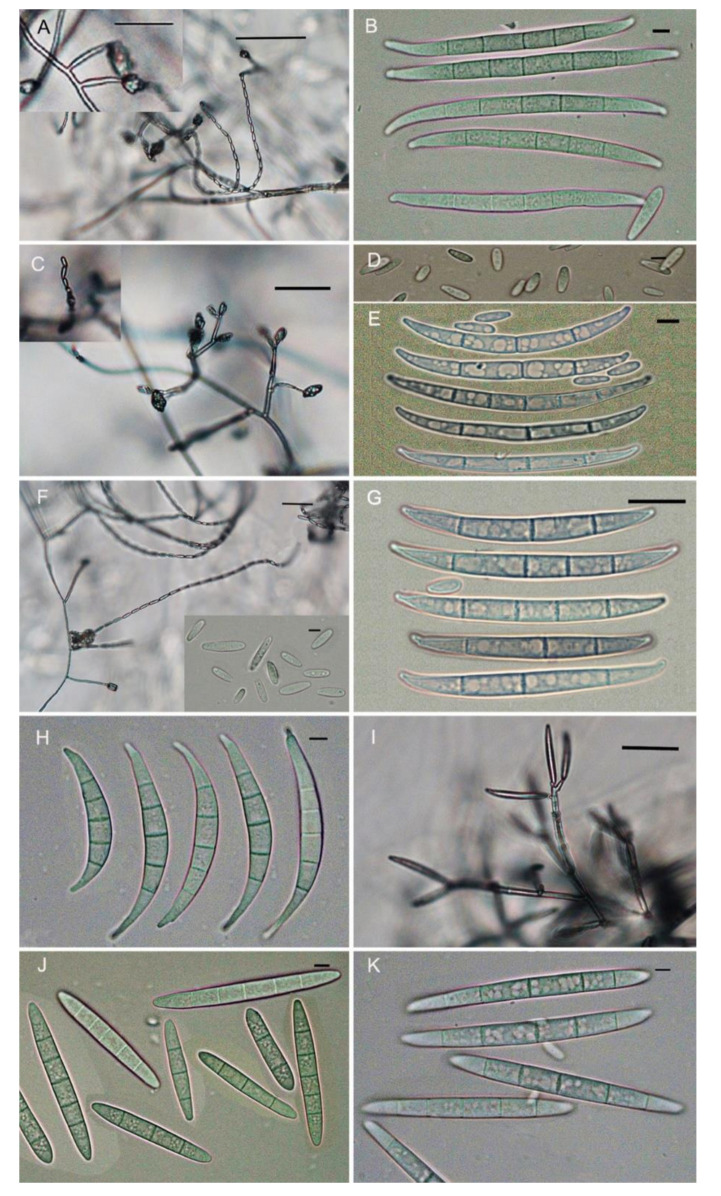
Morphological characteristics of six species of *Fusarium* grown on carnation leaf-piece agar (CLA). (**A**,**B**). *F. fujikuroi*. (**C**)–(**E**). *F. proliferatum.* (**F**)–(**G**). *F. andiyazi*. (**H**). *F. equiseti.* (**I)**–(**J**). *F. incarnatum*. (**K**). *F. asiaticum*. Microconidia. Scale bar = 10 μm.

For identification of *Fusarium* spp., variations of morphological characteristics from all isolates grown on CLA and PDA were compared based on their characteristics and divided into different species complex. Furthermore, the isolates were classified into the FFSC, when macroconidia produced by isolates were relatively slender with no significant curvature, and chlamydospores were absent. In this species complex, microconidia were abundant in false heads or shorter chains (Figure 1A), and macroconidia were relatively slender, medium length with no significant curvature, 3- to 5-septate, apical cell tapered, and basal cell poorly developed (Figure 1B), and they were identified as *F. fujikuroi*. The 337 isolates fell into this species. If microconidia were abundant, formed in chains and in false heads from polyphialides and monophialides (Figure 1C), and were club-shaped, 0-septate (Figure 1D); macroconidia were slender, relatively straight, 3- to 5-septate, and their apical cells were curved and basal cells poorly developed (Figure 1E), and they were identified as *F. proliferatum*. The 35 isolates were attributed to this species. Similarly, if microconidia were clavate to oval with a flattened base, 0-septate and produced in abundance in long chains from monophialides (Figure 1G), macroconidia were straight to slightly curved, and apical cell was slightly curved and basal cell was pedicillate, 3- to 6-septate, mostly 3-septate (Figure 1F) and pseudochlamydospores were formed on this medium, they were identified as *F. andiyazi*. Four isolates belonged to this species, as described by Leslie [28] and Summerell [38]. Macroconidia produced by isolates were the pronounced dorsiventral curvature or falcate [20,28], and they were attributed to the FIESC. In this species complex, if the macroconidia from the sporodochia had pronounced dorsiventral curvature and their apical cells were more rounded comparing with *F. compactum* with the needle-like apical cells (Figure 1H), and there were no microconidia on PDA, as described by Burgess [39], they were identified as *F. equiseti*. The 25 isolates were divided into this species. If the abundant conidia were produced from polyphialides in the aerial mycelia with “rabbit ears” appearance instead of in sporodochia (Figure 1I), and they were straight, spindle-shaped, with 3-4 septa (Figure 1J), conidia also were known as mesoconidia [40]; 11 isolates producing mesoconidia were identified as *F. incarnatum*, which also was used as the more widely recognized name of *F. semitectum* [28]. Macroconidia produced by isolates were relatively slender, curved to almost straight, usually 5-septate, with a curved apical cell and a foot-shaped basal cell, and these isolates were attributed to the FSSC. In this species complex, if macroconidia were straight to slightly curved, 5-septate, apical cell slightly curved, and well-developed basal cell (Figure 1K), very similar to that of *F. graminearum*, they were identified as *F. asiaticum* [37,41]. Two isolates were attributed to this species.

For confirming the results of morphological identification, 421 single-spore isolates were sequenced. Analysis of TEF1-α gene sequences revealed the 378 isolates had 99.9–100% identities with *F. fujikuroi* (337), *F. asiaticum* (2), *F. proliferatum* (35), and *F. andiyazi* (4), respectively, and they could clearly be distinguished by BLAST search, being identical to morphological identification. However, the 36 isolates had 99.9–100% similarity with *F. equiseti* or *F. incarnatum*, and apparently, two species were distinct, even though two species had a difference of significantly morphological characteristics (Figure 1H–J) (Supplementary Table S3). Based on the sequence analysis, the other seven isolates with poor sporulation had 100% similarity with *F. commune* NT-LH01and identified as having *F. commune* (GenBank access No. MF150040.1). Among them, isolate ZJG 17 was deposited in GenBank sequence database (GenBank access No. MT560649).

### 3.2. Diversity of Fusarium spp. on Rice Seeds

Based on the results of identification using combination of morphological and molecular data, 414 isolates were initially divided into six *Fusarium* species (including *F. fujikuroi*, *F. proliferatum*, *F. equiseti*, *F. asiaticum*, *F. incarnatum*, and *F. andiyazi*), while another seven isolates were assigned to *F. commune* only based on TEF1-α gene sequences. According to results of isolation from three provinces (Supplementary Table S4), dominant species with the highest frequency were *F. fujikuroi* (80.05%), and the other species with low frequency in turn were *F. proliferatum* (8.31%), *F. equiseti* (5.94%), *F. incarnatum* (2.61%), *F. commune* (1.66%), *F. andiyazi* (0.95%), and *F. asiaticum* (0.48%). In comparison, seven isolates of *F. commune* were found only on cv. Xiushui 14 from Zhejiang region, and, obviously, they were not representative. The number and origin of these *Fusarium* spp. isolates are summarized in Supplementary Table S4.

### 3.3. Phylogenetic Studies

**Figure 2:**
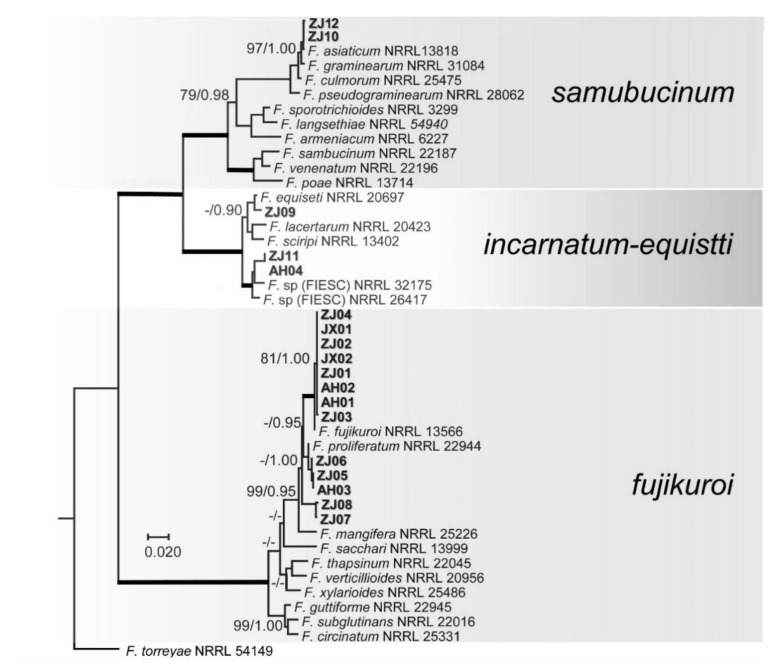
Maximum likelihood (ML) tree obtained from the combined RNA polymerase I largest subunit (RPB1) and RPB2 sequences of 44 taxa of Fusarium. The tree is rooted with *F. torreyae*. Clades with 100% ML bootstrap branch support and 1.00 Bayesian posterior probabilities (BPP) are indicated by thick black lines. Clades with >80% ML_bootstrap (BS) (left values) and 0.95 BPP (right values) are indicated by the corresponding support values. Dashes indicate support values lower than 80% ML-BS and 0.95 BPP. Isolates in this study are shown in bold.

According to geographical location and cultivation area of varieties, 18 representative isolates were selected for PCR amplification (Table 1). Approximately 1500 bp were amplified for RPB1 gene, 900 bp for RPB2, 730 bp for TEF1-α, and 500 bp for ITS region. The first analysis based on *RPB1* and *RPB2* sequences was conducted to evaluate the preliminary identification of the isolates and their phylogenetic affinity among the different species complexes of *Fusarium*. For ML analyses, a GTR + I + G model was selected for all two gene regions and incorporated into the analyses. The combined *RPB1* and *RPB2* sequences dataset included 43 in group taxa with 18 test isolates and *F. torreyae* 54149 as outgroup taxon. The phylogenetic analyses showed that the 18 test isolates were distributed in three *Fusarium* species complexes, namely the FFSC (*fujikuroi*, 13 isolates), FIESC (incarnatum-equiseti, three isolates), and FSSC (*sambucinum*, two isolate) (Figure 2).

**Figure 3:**
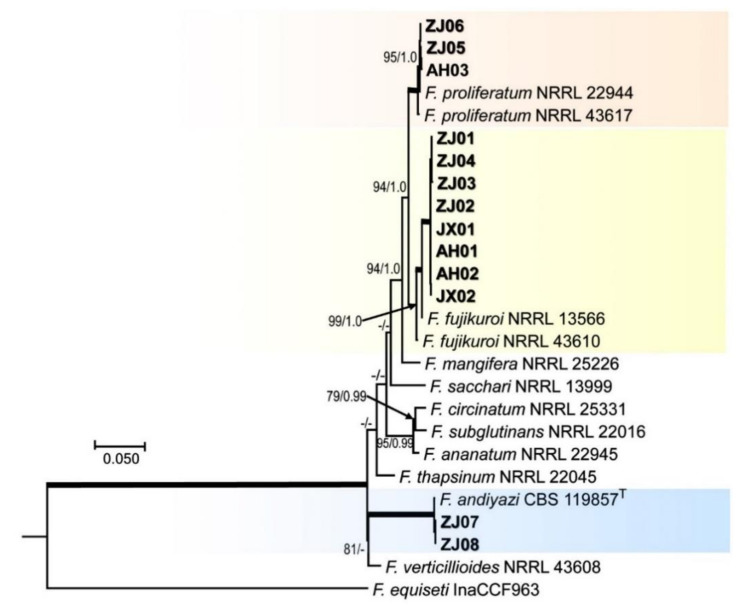
Maximum likelihood (ML) tree obtained from the combined RPB1, RPB2, and translation elongation factor 1-alpha (TEF1)-α sequences of 26 taxa of Fusarium. The tree is rooted with *F. equiseti.* Clades with 100% ML bootstrap branch support and 1.00 Bayesian posterior probabilities (BPP) are indicated by thick black lines. Clades with >80% ML_BS (left values) and 0.95 BPP (right values) are indicated by the corresponding support values. Dashes indicate support values lower than 80% ML-BS and 0.95 BPP. Isolates in this study are shown in bold. T: type species.

To describe the species boundaries of FFSC, phylogenetic tree was conducted by using the three-locus combined RPB1, RPB2, and TEF1-α sequence dataset with 26 taxa. The phylogenetic analyses showed that the three test isolates ZJ05, ZJ06, and AH03 and reference *F. proliferatum* 2294 clustered as a clade, forming a sister clade with reference *F. proliferatum* 43617 with higher ML-BS (100%) and high BI-PP (1.00) support (Figure 3). The eight test isolates (ZJ 01-04, JX01-02, and AH01-02) clustered with reference *F. fujikuroi* 13566 as a clade, forming a sister clade with reference *F. fujikuroi* 43610. Similarly, the two test isolates ZJ07 and ZJ08 clustered with reference *F. andiyazi* 119857 as a clade (Figure 3).

**Figure 4:**
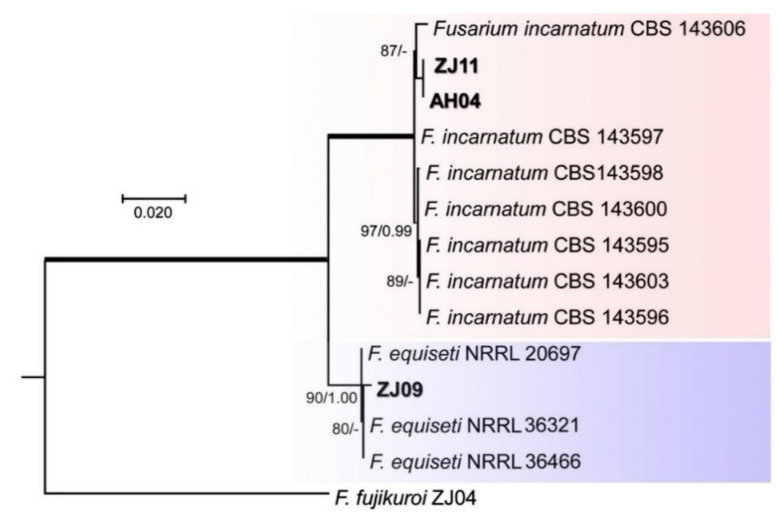
Maximum likelihood (ML) tree obtained from the combined ITS, RBP2, TEF1-α sequences of 14 taxa of *Fusarium*. The tree is rooted with *F. fujikuroi*. Clades with 100% ML bootstrap branch support and 1.00 Bayesian posterior probabilities (BPP) are indicated by thick black lines. Clades with >80% ML_BS (left values) and 0.95 BPP (right values) are indicated by the corresponding support values. Dashes indicate support values lower than 80% ML-BS and 0.95 BPP. Isolates in this study are shown in bold.

For the FIESC, phylogenetic tree constructed by the combination of ITS, RBP2, TEF1-α sequences with 14 taxa dataset showed that the isolate ZJ11 and AH04 clustered with *F. incarnatum* CBS (Centraalbureau voor Schimmelcultures) 143597, 143598, 143600, 143595,143603, and 143596 as a distinct clade with higher ML-BS (87%) and high BI-PP (0.65) support (Figure 4), while the isolate ZJ09 cultured with *F. equiseti* 20697, 36321, and 36466 formed a distinct clade with high ML-BS (90%) and high BI-PP (1.00) support (Figure 4).

**Figure 5:**
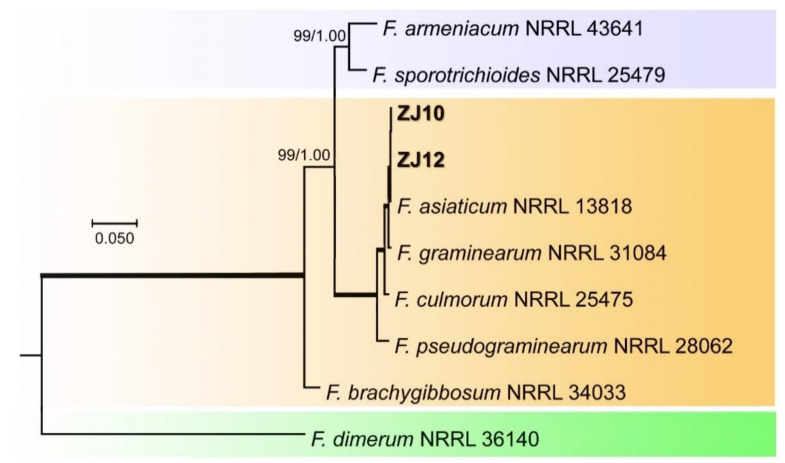
Maximum likelihood (ML) tree obtained from the combined *ITS*, *RBP2*, *TEF1-*α sequences of 10 taxa of *Fusarium*. The tree is rooted with *F. fujikuroi*. Clades with 100% ML bootstrap branch support and 1.00 Bayesian posterior probabilities (BPP) are indicated by thick black lines. Clades with >80% ML_BS (left values) and 0.95 BPP (right values) are indicated by the corresponding support values. Dashes indicate support values lower than 80% ML-BS and 0.95 BPP. Isolates in this study are shown in bold.

Similarly, for analyses of the FSSC, phylogenetic analysis was conducted by using the three-locus combined RPB1, RPB2, and TEF1-α sequence dataset with 10 taxa, including two isolates test ZJ10 and ZJ12 taxa, and *F. dimerum* 36140 was used as outgroup. Phylogenetic analysis showed that the two test isolates ZJ10 and ZJ12 clustered with *F. asiaticum* 13818 as a distinct clade with higher ML-BS (100%) and high BI-PP (1.00) support (Figure 5).

### 3.4. Pathogenicity of Fusarium spp.

**Figure 6:**
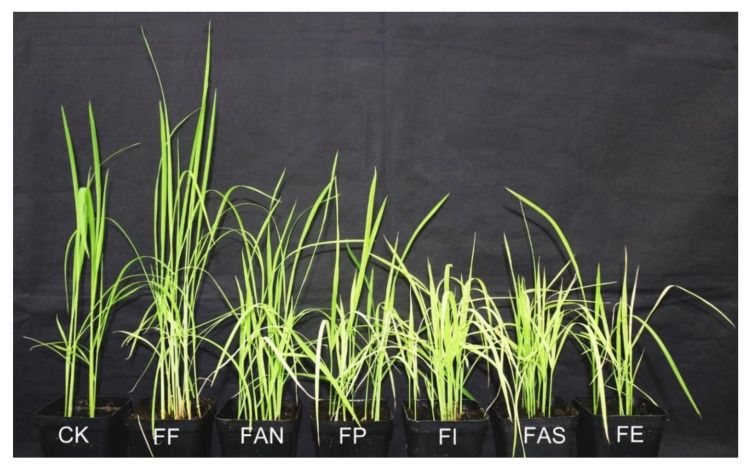
Pathogenicity of different *Fusarium* spp. on rice seedlings 20 days after inoculation. CK: Control; FF: *F. fujikuroi* ZJ01; FAN: *F. andiyazi* ZJ08 FP: *F. proliferatum* ZJ05; FI: *F. incarnatum* ZJ11; FAS: *F. asiaticum* ZJ10; FE: *F. equiseti* ZJ09.

The isolates from six species were used for pathogenicity tests with inoculated seeds to evaluate the virulence differences between different *Fusarium* species. After fifteen days of sowing, symptoms of bakanae disease was observed on seedlings inoculated with *F. fujikuroi* ZJ01, while individual yellowing leaves appeared on seedlings inoculated with *F. andiyazi* ZJ08 and *F. proliferatum* ZJ05, and relative stunted plants with yellowing leaves were observed on seedlings inoculated with *F. incarnatum* ZJ11, *F. asiaticum* ZJ10, and *F. equiseti* ZJ09. However, no *symptoms were observed* on *sterile water-inoculated* seedling *controls. After twenty days of sowing,* typical bakanae disease symptoms were observed on seedlings inoculated with *F. fujikuroi* ZJ01 (Figure 6), as described by Choi [9]. The yellowing leaves, including few dying leaf/leaves, were observed on normal height seedlings inoculated with *F. andiyazi* ZJ08 and *F. proliferatum* ZJ05. In comparison, the yellowing leaves, including few dying leaves, Fwere *visible on stunted seedlings inoculated with* *F. incarnatum* ZJ11, *F. asiaticum* ZJ10, and *F. equiseti* ZJ09. The results revealed that isolates of six species were all pathogens and able to cause disease, but there were differences in virulence.

In vitro seed germination tests were conducted using inoculated seeds to reveal the reason for differences in virulence among *Fusarium* species. The test results showed that the isolates of six *Fusarium* species all influenced germination of rice seeds, including complete inhibition of seed germination (non-germinated), seed and sprouts decay, as well as seedling stunt ten days after inoculation (Supplementary Figure S3). Inoculation increased percentage inhibition (PI) of seeds germination by 73% in *F. asiaticum* ZJ10, 71% in *F. andiyazi* ZJ08, 54% in *F. incarnatum* ZJ11, 44% in *F. equiseti* ZJ09, and 35% in *F. proliferatum* ZJ05, respectively, but there was no significant difference between *F. asiaticum* ZJ10, and *F. andiyazi* ZJ08, as well as among *F. incarnatum* ZJ11, *F. proliferatum* ZJ05, and *F. equiseti* ZJ09 (*p* < 0.05). The repeated tests also showed that the seed germination PI value was almost the same as the above result (Supplementary Table S5). In addition, observation of symptoms indicated that small dark spots with the size of a pinpoint appeared first at the top of a sprout three days after inoculation. Subsequently, clear sprout decay symptoms were observed at 5–7 days, as shown in Supplementary Figure S3. After ten days, *F. andiyazi* ZJ08 inoculation showed the highest percentage of sprouts decay (PSD) by 68.8 ± 8.5%, followed by PSD by 62.5 ± 8.7% in *F. asiaticum* ZJ10, 50.0 ± 4.1% in *F. incarnatum* ZJ11, 37.5 ± 9.6% in *F. equiseti* ZJ09, 28.8 ± 6.3% in *F. proliferatum* ZJ05, and 27.5 ± 6.5% in *F. fujikuroi* ZJ01, respectively. Still, there was no significant difference between *F. andiyazi* ZJ08 and *F. asiaticum* and among *F. fujikuroi* ZJ01, *F. proliferatum* ZJ05, and *F. equiseti* ZJ09 (*p* < 0.05) (Supplementary Table S5). Although the inoculation with *F. proliferatum* ZJ05 gave the greatest percentage of non-germinated seeds (PUS) by 17.5 ± 4.3%, followed by PUS by 16.3 ± 6.5% in *F. equiseti* ZJ09, 8.8 ± 6.5% in *F. asiaticum* ZJ10, 8.8 ± 5.4% in *F. andiyazi* ZJ08, 6.3 ± 5.4% in *F. fujikuroi* ZJ01, and 2.5 ± 2.5% in *F. incarnatum* ZJ11, respectively, there was no significant difference was observed between *F. proliferatum* ZJ05 and *F. equiseti* ZJ09 and among *F. andiyazi* ZJ08, *F. asiaticum*, *F. fujikuroi* ZJ01, and *F. incarnatum* ZJ11 (*p* < 0.05) (Supplementary Table S5).

### 3.5. LAMP Primers

The promoters and coding sequences analysis of enzyme-coding genes in gibberellins biosynthesis pathway in *Fusarium* spp. showed that the promoter sequences of desaturase gene (des) in *F. fujikuroi* (Supplementary sequence 1) had 81.26% identity with *F. proliferatum* (GenBank access No. AJ628021) instead of no similarity with other *Fusarium*. spp. in the GenBank database. Therefore, according to tis conserved region of the promoter sequences (Supplementary sequence 2), the primer pair DesF/DesR was designed, and they were used for PCR amplification from 23 isolates of *Fusarium* spp. and other plant fungal pathogens (Supplementary Table S2).

The PCR products were observed only in the isolates of *F. fujikuroi*, *F. proliferatum*, and *F. oxysporum*. The sequence analysis showed that the promoter sequences from *F. fujikuroi* had a relatively higher similarity with *F. proliferatum* than *F. oxysporum*. A 231 bp sequence was selected from promoter region of the desaturase-coding gene of *F. fujikuroi* as the marker (Supplementary Figure S6), which was designated as DesM_231_, and LAMP primers were designed by the Primer Explorer V5 software based on DesM_231_ sequence. The location and direction of primers are indicated in the Supplementary Figure S6. The primer sequences are shown in Table 2.

### 3.6. LAMP Reaction and Optimization of Its Conditions

The LAMP assay was carried out using *F. fujikuroi* DNA as a template at 50, 55, 60, 65, 67, 68, 69, and 70 °C, respectively, for 55 min. Although LAMP products were observed from 55 to 68 °C for 55 min according to color change (visual inspection) and electrophoresis on agarose, the best results were obtained when the reaction temperature was maintained at 65 °C (Supplementary Figure S7A,B).

Furthermore, the LMAP test was carried out at 65 °C for different times, including 25, 30, 35, 40, 45, 50, 55, 60, 65, 70, and 75 min. The results showed that LAMP products turned to green color in tubes treated from 25 to 75 min, and only tubers used as control and treated for 20 min turned to orange (Supplementary Figure S8A); however, the best results were produced after reaction time was maintained for 60 min based on electrophoresis (Supplementary Figure S8B).

### 3.7. Specificity and Sensitivity of LAMP Reaction

**Figure 7:**
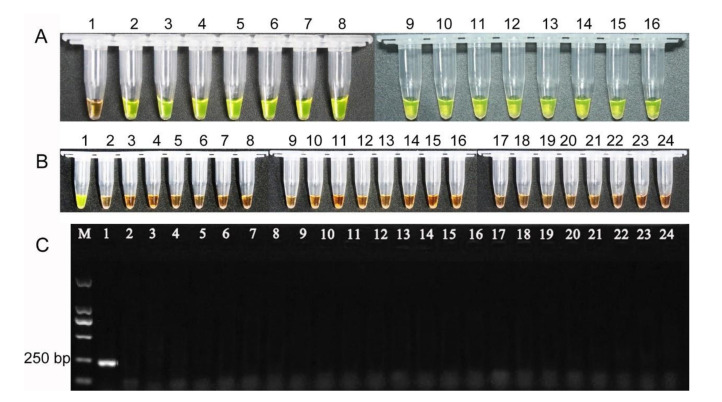
Analysis of reliability and specificity of LAMP assays. (**A**). Detection of *F. fujikuroi* isolates from different origins. The tubes from 2 to 15 show the LAMP products amplified from *F. fujikuroi* isolate. Tuber 1 is used as control without template DNA. B. Specificity of LAMP assays. Tube 1–24 contain the DNA template originating from isolates of *Fusarium fujikuroi*, *F. proliferatum*, *F. equiseti*, *F.* *oxysporum*, *F. avenaceum*, *F. asiaticum*, *F. graminearum*, *F. solani*, *F. verticillioides*, *F. incarnatum*, *F. commune*, *F. Andiyazi*, *F. Boothii*, *Ustilago esculenta*, *Verticillium dahlia* 086, *V. dahlia* BP2, *Pyricularia oryzae*, *Ustilaginoidea oryzae,* Sclerotinia sclerotiorum, *Phytophthora capsica*, *Scopulariopsis* *gossypii*, and *P. nicotianae*, respectively (Supplementary Table S2). (**B**). Electrophoretogram of PCR products. Fungal isolates and their DNA templates are the same as Figure B. (**C**) Electrophoretogram of PCR products. Lanes 1–24: contain the DNA template originating from isolates of *Fusarium fujikuroi*, *F. proliferatum*, *F. equiseti*, *F.* *oxysporum*, *F. avenaceum*, *F. asiaticum*, *F. graminearum*, *F. solani*, *F. verticillioides*, *F. incarnatum*, *F. commune*, *F. Andiyazi*, *F. Boothii*, *Ustilago esculenta*, *Verticillium dahlia* 086, *V. dahlia* BP2, *Pyricularia oryzae*, *Ustilaginoidea oryzae,* Sclerotinia sclerotiorum, *Phytophthora capsica*, *Scopulariopsis* *gossypii*, and *P. nicotianae*, respectively (Supplementary Table S2), respectively. CK: control; M: DNA marker.

The LAMP assay was conducted using the15 isolates of *F. fujikuroi* at 65 °C for 60 min, and amplification products were observed by visual inspection in all 15 isolates (Figure 7A), showing reliability of primers. To confirm LAMP assay specificity, the 23 isolates of *Fusarium* spp. and other plant fungal pathogens (Supplementary Table S2) were used as the LAMP assay, which was conducted at 65 °C for 60 min. The results demonstrated that LAMP products turned to green color only in *F. fujikuroi* ZJ01 and turned to orange in other isolates (Figure 7B), showing that LAMP primers were species-specific. Meanwhile, the results of PCR amplification also displayed that PCR product was observed only in *F. fujikuroi* ZJ01 (Figure 7C), further confirming the results of the LAMP assay.

**Figure 8:**
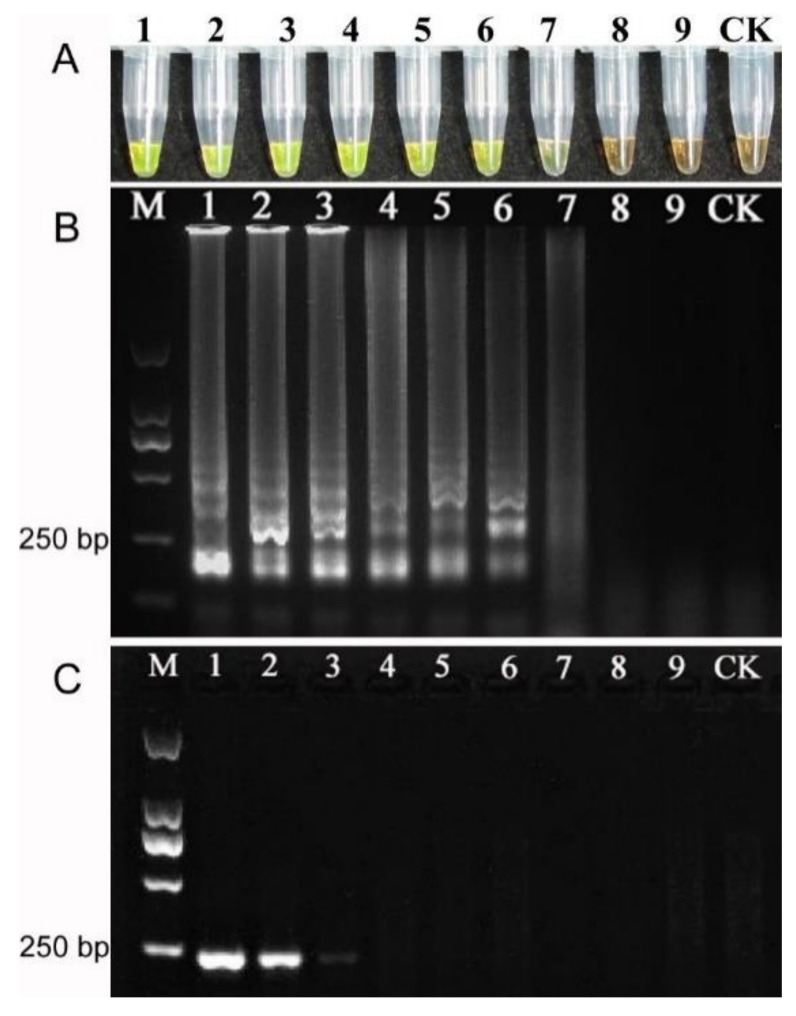
Comparative sensitivity of LAMP and PCR reaction for detection of *F*. *Fujikuroi* ZJ01 using different DNA template concentration. (**A**). Visual inspection of LAMP reaction using SYBR Green I dye. Tubes 1–9: DNA template concentration of 9.40 ng, 0.94 ng, 94.00 pg, 9.40 pg, 0.94 pg, 94.00 fg, 9.40 fg, 0.94 fg, 94.00 ag, respectively. (**B**). Electrophoretogram of LAMP products. Lanes 1–9 are corresponding to the tubes 1–9 in Figure (**C**). Electrophoretogram of PCR products. Lanes 1–9: DNA template concentration of 9.40 ng, 0.94 ng, 94.00 pg, 9.40 pg, 0.94 pg, 94.00 fg, 9.40 fg, 0.94 fg, 94.00 ag, respectively. CK: control; M: DNA marker.

For verifying the sensitivity of LAMP assay, genomic DNA extracted from *F*. *Fujikuroi* ZJ01 with concentration of 9.4 ng, 0.94 ng, 94 pg, 9.4 pg, 0.94 pg, 94 fg, 9.4 fg, 0.94 fg, 94 ag, respectively, was used as DNA template for LAMP and PCR amplification at 65 °C for 60 min. The results of LAMP amplification revealed that about 9.4 fg/μL template was sufficient for LAMP-based diagnostics (Figure 8A,B). In comparison, approximate 94 pg/μL template was the lowest concentration for conventional PCR detection (Figure 8C) and showed that this detection limit was 10,000-times lower than that of LAMP amplification.

### 3.8. Detection of Rice Seed Samples

**Figure 9:**
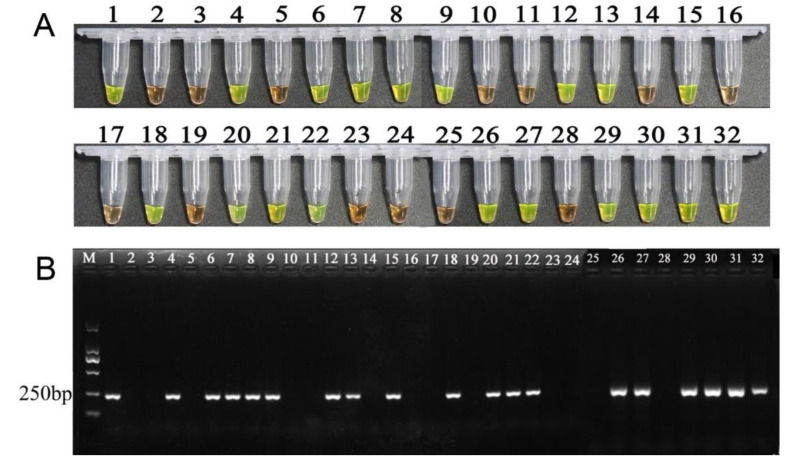
Detection of LAMP amplification for *F. fujikuroi* in rice seed samples. (**A**). Visual inspection of LAMP reaction using SYBR Green I dye. LAMP reactions in tubes 1, 4, 6–9,12, 13, 15, 18, 20–22, 26, 27, and 29–32 were conducted from 18 diseased rice seed samples. LAMP reactions in tubes 3, 5, 10–11, 14, 16–17, 19, 23–25, and 28 were conducted from 12 healthy rice seed samples. Tube 1: positive control (with DNA template of *F. fujikuroi*); Tube 2: negative control (without DNA template). (**B**). Electrophoretogram of PCR products. Serial number of lanes corresponds with the number of tubers in Figure A. M: DNA mark.

The LAMP assay was used for the detection of *F*. *fujikuroi* in natural seeds. The results of LAMP amplification showed that LAMP products amplifying from 18 samples of diseased rice seeds all turned to green color (Figure 9A), while, from 12 samples of healthy rice seeds, all turned to orange, displaying the reliability of LAMP reaction. Similarly, the PCR amplification results using DNA template of the same concentration for each sample demonstrated that PCR products were found only in 18 samples of diseased but no PCR product in 12 samples of healthy rice seed samples (Figure 9B).

## 4. Discussion

### 4.1. Diversity of Fusarium spp. on Rice Seeds

This is the first study to systematically analyze the *Fusarium* species on rice seed from three provinces of China. The identification of the *Fusarium* species revealed that 89.31% of the isolates belong to the FFSC, showing them to be dominant that is similar to previous studies [4,9,42]. Among the FFSC, although four species (*F. andiyazi*, *F. fujikuroi*, *F. proliferatum* and *F. verticillioides*) were deemed to be associated with RBD [8], *F. verticillioides* was not found in this study. In addition, another four species with low isolation frequency in this study, belong to FIESC (*F. incarnatum* and *F. equiseti*), FSSC (*F. asiaticum*), and *F. oxysporum* species complex (FOSC; *F. commune*) [43], were also found on rice seeds in other countries [4,9,20]. However, *F. commune* was found in only one variety that was isolated in the group.

The isolation frequency and species variation on rice seeds depend on the origin (different countries, regions, and varieties), showing diversity of *Fusarium* spp. However, differences in species composition between studies may be due to various factors, e.g., sample size, target organ (plant or seed), geographical distribution, and climate effect of species distribution [44]. In China, rice is one of the prominent cereal crops, and about 65% of Chinese people rely on rice. Although this survey of the *Fusarium* species has its limitations in geographical range and number of varieties in rice-growing regions of China, our results provide an important information for nationwide survey of diversity of the *Fusarium* species in future.

### 4.2. Identification of Fusarium spp.

The identification of *Fusarium* spp. associated with bakanae disease only depending on morphological characteristics is a highly challenging task due to its diverse characteristics [9]. Thus, standardized culture media and methods, along with multi-locus molecular data, are currently required for confident identification at the species level. Although some studies have demonstrated that the FFSC associated with bakanae, including other several species (*F. thapsinum*, *F. pseudonygamai*, *F. moniliforme*, and *F. commune*), can be clearly distinguished by only the TEF1-α gene sequence [8,9,41], no evidence showed that TEF1-α gene was suitable to delimitation of all *Fusarium* species. However, a combination of RPB1 and RPB2 genes were used to infer evolutionary relationships of *Fusarium* species complex [7], while combination of RPB1, RPB2 and TEF1-α genes even were used for genetic diversity analysis of *Fusarium oxysporum* f. sp. Cubense [45] and especially recommended [30]. However, due to lacking available reference sequences of RPB1 gene in *F. incarnatum*, six species identified by morphology and TEF1-α gene in this study, wherefore, were first divided into the species complex only using RPB1 and RPB2 genes due to lacking references sequences of individual species (Figure 2), and then each species complex was analyzed further using different loci sequences. In the FFSC, morphologically microconidia of *F. fujikuroi* forms are oval or club-shaped with a flattened base having 0 to 1 septum, and false heads and chains of short to medium length are produced from poly- and monophialides [28]. The macroconidia are relatively slender with tapering apical, and poorly developed basal cell with 3 to 5 septa and chlamydospores are absent. Especially, *F. fujikuroi* has very similar morphological characteristics with *F. proliferatum*. Phylogenetically, *F. fujikuroi* and *F. proliferatum* are closely related species. In the FIESC, species belonging to the incarnatum or equiseti morphotype morphologically have been described by Avila [20], although morphological markers cannot distinguish most of the cryptic species in the FIESC. In the FSSC, *F. andiyazi* is morphologically very similar to *F. verticillioides*, and the difference is that *F. andiyazi* produces pseudo-chlamydospores only in this species [28], and their close relationship also was confirmed by phylogenetic analysis, as showed in Figure 2. In the FSSC, *F. asiaticum* is morphologically indistinguishable from *F. graminearum, and they* have slightly different conidial features [40], but phylogenetic analysis supports their species delimitation, as showed in Figure 2. Our results confirmed that RPB1, RPB2, and TEF1-α genes are reliable molecular markers similar to other studies [30,44]. This will provide an important information for identification of *Fusarium* spp. on rice seed.

In addition, due to the fact that the relationship of each SC (species complex) with corresponding secondary metabolites has been established by Reference [7,20], mycotosins, like trichothecene in the FSSC and FIESC and fumonisins in FFSC, could be predicted, which provided available clues for analyzing pathogenicity of *Fusarium* spp.

### 4.3. Pathogenicity of Fusarium spp.

*F. fujikuroi* isolates were able to cause bakanae disease, and other species also have been studied [2,3]. However, various studies have found that the FFSC (including *F. proliferatum*, *F. verticillioides*, and *F. andiyazi*) were associated with the bakanae disease on rice [8,9,46,47]. In this study, isolate of six species were capable of causing symptoms inherent to bakanae, but the difference of symptoms was significant (Figure 6). The isolate of *F. fujikuroi* promoted the elongation of seedlings, isolate of *F. andiyazi*, and *F. proliferatum* caused the relative stunting of seedlings comparing with control, being consistent with other reported findings [8,41]. Interestingly, the severe stunting occurred in seedlings inoculated with isolates of *F. equiseti*, *F. asiaticum*, and *F. incarnatum*, respectively. Although *F. concentricum* is capable of causing symptoms of RBD [48], and *F. incarnatum* (*F. semitectum*) is a pathogen of bakanae [49], *F. equiseti* is known as a saprophytic colonizer [9], while the role of *F. asiaticum* as a saprophytic or pathogenic fungus has not been documented. In vitro tests of seed germination were conducted to reveal the reason for different symptoms in pathogenicity. The results showed that six species isolate all influenced rice seed germination (Supplementary Figure S3). The isolates of *F. asiaticum* and *F. andiyazi* caused the highest percentage inhibition of seed germination, followed by *F. equiseti*, *F. incarnatum*, *F. proliferatum*, and *F. fujikuroi* (Supplementary Figure S4). The inhibitory mechanism of seed germination is related to mycotoxins.

In the FFSC, F. fujikuroi is known to produce Gibberellin A3 only in this species, which promotes elongation of plants, as shown in Figure 6. FFSC isolates produce at least one of the mycotoxins (beauvericin (BEA), fumonisins (FBs), moniliformin (MON), and enniatins (ENNs)) [9]. Although fumonisins, moniliformin, and fusaric acid were confirmed to be involved in phytotoxicity in plants and express of symptoms [4,50,51], there was no available information about their effect on rice seed germination. In the FIESC and FSSC, FIESC isolates from rice seeds are capable of producing trichothecenes, including deoxynivalenol (DON), nivalenol (NIV), 4-acetylnivalenol (4-ANIV), 15-acetyldeoxynivalenol (15-ADON), and 3-acetyldeoxynivalenol (3-ADON) [20]. Another study finds that F. equiseti produces the T-2 toxin except for DON [52]. However, most F. asiaticum isolates from Jiangsu province of China, belonging to FSSC, mainly produce 3-acetyldeoxynivalenol (3-ADON) and the remainder produce nivalenol (NIV) [53]. Many trichothecenes are phytotoxic [54], and some, such as 4,15-Diacetoxyscirpenol (DAS), have been shown to inhibit seed germination of soybean [55]. In this study, our results provided new evidences that F. asiaticum, F. equiseti, and F. incarnatum were also pathogens, which inhibited seed germination and caused seed and bud rot; however, it is unclear which derivative of trichothecenes plays a leading role in inhibiting rice seed germination.

### 4.4. Application of LAMP as Diagnostic Tool

The LAMP method possesses the advantage of fast, sensitive, reliable, and inexpensive technology over PCR and is widely used as diagnostic tool. However, sensitivity of assay involves the design of suitable primers and the optimization of reaction conditions, while specificity of LAMP assay depends on the uniqueness of the target marker. In detection of *F. fujikuroi*, for example, although the TEF1 gene [23] and intergenic spacer (IGS) region [36] are used as markers, successful LAMP amplification requires relatively strict reaction conditions due to high similarity of marker sequences. In comparison, sequences of unique or very different fragments are used for LAMP primer design, and reliable results are obtained easily. In this study, the promoter sequence of desaturase in Gibberellins biosynthesis pathway was used as the target marker (DesM231) due to difference of larger bases between *F. fujikuroi* and other species of *Fusarium* (Supplementary Figure S6). LAMP amplification was observed under various conditions, as described above, and the sensitivity of LAMP reaction was increased for more than 9.4 fg under an optimization condition, and assays for detecting rice seeds from the fields further confirm that the developed LAMP technique using for detection of *F*. *fujikuroi* is reliable. In addition, LAMP amplification using DesM231 marker provides information related to GA3 production (from its precursors GA4 and GA7) (Supplementary Figure S5). Although the non-ribosomal peptide synthetase gene (NRPS31) in *F. fujikuroi*, consisting of 11-gene cluster (apicidin synthetase, APS) without production of apicidin [56], is used as a marker in LAMP assays [6,25], NRPS31 is closely related to the NRPS APS1 (66–71% identical) in *F. incarnatum*, which is part of a 12-gene cluster responsible for synthesis of apicidin, a histone deacetylase inhibitor with antiparasitic activity [57,58], showing that it is not unique in genus *Fusarium*. Furthermore, a previous study showed that *F. fujikuroi* m567 (Genbank accession no. AJ417493) contained all of the GA-biosynthesis genes and produced GA3 [18]. In comparison, its mutant analysis revealed that GA4 to GA7 and GA7 to GA3 were converted by the 13-hydroxylase, respectively. In this study, although GA3 production is not detected, pathogenicity tests provide indirect evidence and sequences of desaturase promoters of *F. fujikuroi* having 99.88% identities with *F. fujikuroi* m567. Therefore, GA3 production could be anticipative in *F. fujikuroi* isolates. This indicates that LAMP-based detection developed and standardized for the detection of *F. fujikuroi* associated with RBD on seeds is a powerful tool for the rapid and sensitive detection of *F. fujikuroi* and predicting GA3 production.

## Figures and Tables

**Table 1:** GenBank accessions No. of *Fusarium* isolates used for phylogenetic analysis in this study.

Species	Isolates	Origin	GenBank Access NO.			
			TEF1-α RPB1 RPB2 ITS			
*Fusarium fujikuroi*	ZJ01	Zhejiang China	MT560637	MT560601	MT560616	
*Fusarium fujikuroi*	ZJ02	Zhejiang China	MT560638	MT560602	MT560617	
*Fusarium fujikuroi*	ZJ03	Zhejiang China	MT560639	MT560603	MT560618	
*Fusarium fujikuroi*	ZJ04	Zhejiang China	MT560640	MT560604	MT560619	
*Fusarium fujikuroi*	AH01	Anhui China	MT560650	MT560611	MT560628	
*Fusarium fujikuroi*	AH02	Anhui China	MT560651	MT560612	MT560629	
*Fusarium fujikuroi*	JX01	Jiangxi China	MT560654	MT560614	MT560632	
*Fusarium fujikuroi*	JX02	Jiangxi China	MT560655	MT560615	MT560633	
*Fusarium proliferatum*	ZJ05	Zhejiang China	MT560641	MT560605	MT560620	
*Fusarium proliferatum*	ZJ06	Zhejiang China	MT560642	MT560606	MT560621	
*Fusarium proliferatum*	AH03	Anhui China	MT560652	MT560613	MT560630	
*Fusarium andiyazi*	ZJ07	Zhejiang China	MT560643	MT560607	MT560622	
*Fusarium andiyazi*	ZJ08	Zhejiang China	MT560644	MT560608	MT560623	
*Fusarium asiaticum*	ZJ10	Zhejiang China	MT560646	MT560609	MT560625	
*Fusarium asiaticum*	ZJ12	Zhejiang China	MT560648	MT560610	MT560627	
*Fusarium equiseti*	ZJ09	Zhejiang China	MT560645		MT560624	MT560634
*Fusarium incarnatum*	ZJ11	Zhejiang China	MT560647		MT560626	MT560635
*Fusarium incarnatum*	AH04	Anhui China	MT560653		MT560631	MT560636
*Fusarium commune*	ZJG17	Zhejiang China	MT560649			
*Fusarium andiyazi*	CBS 119857	Netherlands	KP662901	LT996189	KT154004	
*Fusarium_fujikuroi*	NRRL 13566	Taiwan	AF160279	JX171456	JX171570	
*Fusarium proliferatum*	NRRL 22944	Germany	AF160280	JX171504	HM068352	
*Fusarium_mangifera*	NRRL 25226	India	AF160281	JX171509	JX171622	
*Fusarium_sacchari*	NRRL 13999	India	AF160278	JX171466	JX171580	
*Fusarium_thapsinum*	NRRL 22045	Natal, South Africa	AF160270	JX171487	JX171600	
*Fusarium_subglutinans*	NRRL 22016	IL-USA	AF160289	JX171486	JX171599	
*Fusarium_circinatum*	NRRL 25331	CA-USA	AF160295	JX171510	JX171623	
*Fusarium_ananatum*	NRRL 22945	England	AF160297	JX171505	JX171618	
*Fusarium fujikuroi*	NRRL 43610	Iowa	HM347123	HM347184	EF470199	
*Fusarium verticillioides*	NRRL43608	Minnesota	HM347122	HM347183	EF470197	
*Fusarium proliferatum*	NRRL43617	Colorado	HM347124	HM347185	EF470206	
*Fusarium equiseti*	InaCC F963	Indonesia	LS479445	LS479875	LS479859	
*Fusarium asiaticum*	NRRL 13818	Japan	AF212451	JX171459	JX171573	
*Fusarium graminearum*	NRRL 31084	MI-USA	AY452957	JX171531	JX171644	
*Fusarium culmorum*	NRRL 25475	Denmark	KY873384	JX171515	JX171628	
*Fusarium pseudograminearum*	NRRL 28062	USA	AF212468	JX171524	JX171637	
*Fusarium* sporotrichioides	NRRL 25479	Germany	HM744652	HM347144	HQ154441	
*Fusarium graminearum*	NRRL 43641	Missouri	GQ505430	HM347192	GQ505494	
*Fusarium brachygibbosum*	NRRL 34033	Texas unknown	GQ505418	HM347172	GQ505482	
*Fusarium dimerum*	NRRL36140	Zhejiang China	HM347133	HM347203	HM347218	
*Fusarium incarnatum*	CBS143596	Moghan-Ardabil Iran	LT970779		LT970751	LT970815
*Fusarium incarnatum*	CBS143595	Moghan-Ardabil Iran	LT970778		LT970750	LT970814
*Fusarium incarnatum*	CBS143598	Moghan-Ardabil Iran	LT970780		LT970752	LT970816
*Fusarium incarnatum*	CBS143603	Moghan-Ardabil Iran	LT970782		LT970754	LT970818
*Fusarium incarnatum*	CBS143600	Moghan-Ardabil Iran	LT970781		LT970753	LT970817
*Fusarium incarnatum*	CBS143606	Moghan-Ardabil Iran	LT970783		LT970755	LT970819
*Fusarium incarnatum*	CBS143597	Moghan-Ardabil Iran	LT970784		LT970756	LT970820
*Fusarium equiseti*	NRRL 20697	Chile	GQ505594		GQ505772	GQ505683
*Fusarium equiseti*	NRRL 36321	Netherlands	GQ505647		GQ505825	GQ505736
*Fusarium equiseti*	NRRL 36466	Denmark	GQ505653		GQ505831	GQ505742
*Fusarium_sporotrichioides*	NRRL 3299	WI-USA		JX171444	JX171558	
*Fusarium_langsethiae*	NRRL 54940	Norway		JX171550	JX171662	
*Fusarium_armeniacum*	NRRL 6227	MO-USA		JX171446	JX171560	
*Fusarium_sambucinum*	NRRL 22187	England		JX171493	JX171606	
*Fusarium_venenatum*	NRRL 22196	Germany		JX171494	JX171607	
*Fusarium_poae*	NRRL 13714	Manitoba, Canada		JX171458	JX171572	
*Fusarium_sciripi*	NRRL 13402	NSW, Australia		JX171566	JX171452	
*Fusarium_lacertarum*	NRRL 20423	India		JX171467	JX171581	
*Fusarium_equiseti*	NRRL 20697	Chile		JX171481	JX171595	
*Fusarium_sp._FIESC_26a*	NRRL 26417	Cuba		JX171522	JX171635	
*Fusarium_sp._FIESC_15a*	NRRL 32175	TX-USA		JX171532	JX171645	
*Fusarium_verticillioides*	NRRL 20956	CA-USA		JX171485	JX171598	
*Fusarium_xylarioides*	NRRL 25486	Ivory Coast		JX171517	JX171630	
*Fusarium torreyae*	NRRL 54149	FL-USA		JX171548	JX171660	

**Table 2:** Nucleotide sequences of the loop-mediated isothermal amplification (LAMP) primers designed in this study.

Primer Type	Primer Sequence (5′-3′)	Length
F3	TTGACAAAGTTCGGTGCC	18
B3	TCTGACTTGATTCACAGATG	20
FIP (F1c + F2)	GCTACCAAGGTAAGGTATCTGCTAAGTTGTTTGTCGGCTGATC	43
BIP (B1c + B2)	GCTTCACTGGCCTTGGAGTCATAAGGTTTATGGAGACGCAC	41
LoopF	GGCGTTCATACCACGACCT	19
LoopB	GTACGGTATCACCGTTGCATT	21

## Data Availability

The data presented in this study are available in insert article or supplementary material here.

## Supplementary Materials

The following are available online at https://www.mdpi.com/2076-0817/10/1/1/s1. Figure S1: Geographical location of Zhejiang, Anhui and Jiangxi provinces in China. Red color: Zhejiang province; Yellow: Anhui province; blue: Jiangxi province. Figure S2: Typical symptoms of bakanae on rice seedlings in field. A. Abnormal elongation plants with chlorotic leaves. B. A partial magnification in Figure A. Figure S3: Inhibitory effect of different *Fusarium* spp. on rice seed germination 10 days after inoculation. CK: Control; FF: *F. fujikuroi* ZJ01; FAN: *F. andiyazi* ZJ08; FP: *F. proliferatum* ZJ05; FI: *F. incarnatum* ZJ11; FAS: *F. asiaticum* ZJ10; FE: *F. equiseti* ZJ09. Arrowheads: inhibition of seed germination; arrows: seed rot; double arrows: bud rot. Figure S4: Percentage inhibition of seed germination after inoculation with isolates of *Fusarium fujikuroi* ZJ01, *F. proliferatum* ZJ05, *F. andiyazi* ZJ08*, F. asiaticum* ZJ10, *F. incarnatum* ZJ11 and *F. equiseti* ZJ09 for 10 days. The same letter(s) are not different significantly at *p* < 0.05 according to LSD. Figure S5: Substrate and product of desaturase marked in red in the last step of gibberellin biosynthesis pathways. Figure S6: Partial sequences of the desaturase gene promoters of in *Fusarium fujikuroi* ZJ01and *F. proliferatum* ZJ05 and location of Loop-Mediated Isothermal Amplification (LAMP) primers. Arrows indicate LAMP primers (inner and outer primers), along with their position for detection of *F. fujikuroi*. Figure S7: Detection of *F. fujikuroi* LAMP products under different temperature for 55 min. A. Visual inspection of LAMP reaction using SYBR Green I dye. The tubes from 1 to 8 are maintained at 50, 55, 60, 65, 67, 68, 69, and 70 °C, respectively. B. Electrophoretogram of reaction products. Lanes 1–8 come from tubes 1–8. M, DNA marker. Figure S8: Detection of *F. fujikuroi* LAMP products at 65 °C for different time. A. Visual inspection. The tubes 2–12 are maintained for 20, 25, 30, 35, 40, 45, 50, 55, 60, 65, 70, and 75 min, respectively. Tuber 1: negative control. B. Electrophoretogram of LAMP products. Lanes 2–12, corresponding to tubes 2–12. Lane 1: negative control. M: DNA marker. Table S1: Rice varieties and origin in this study. Table S2: Strains of fungi used for testing the specificity in the loop mediates isothermal amplification (LAMP) and polymerase chain reaction (PCR) assays. Table S3: GenBank accessions No. of 402 Fusarium isolates used for phylogenetic analysis in this study. Table S4: Number and isolation frequency of *Fusarium* species from 25 rice varieties in three provinces. Table S5: The results of rice seed germination tests by inoculation with six Fusarium spp. Isolates. Sequence1: Desaturase Gene from NBCI database. Sequence2: Partial sequences of desaturase promoter region amplified using primer pair DesF/DesR.
