# Supplementary material for: Identification of Rice Seed-Derived Fusarium spp. and Development of LAMP Assay against Fusarium fujikuroi
Source: Pathogens. 2020 Dec 22;10(1):1. doi: 10.3390/pathogens10010001 (PMC7822049; doi:10.3390/pathogens10010001)
Supplement: Supplementary file 1 [file pathogens-10-00001-s001.zip › Supplemtary files/Supplementary Figures/Supplementary Figures.docx]

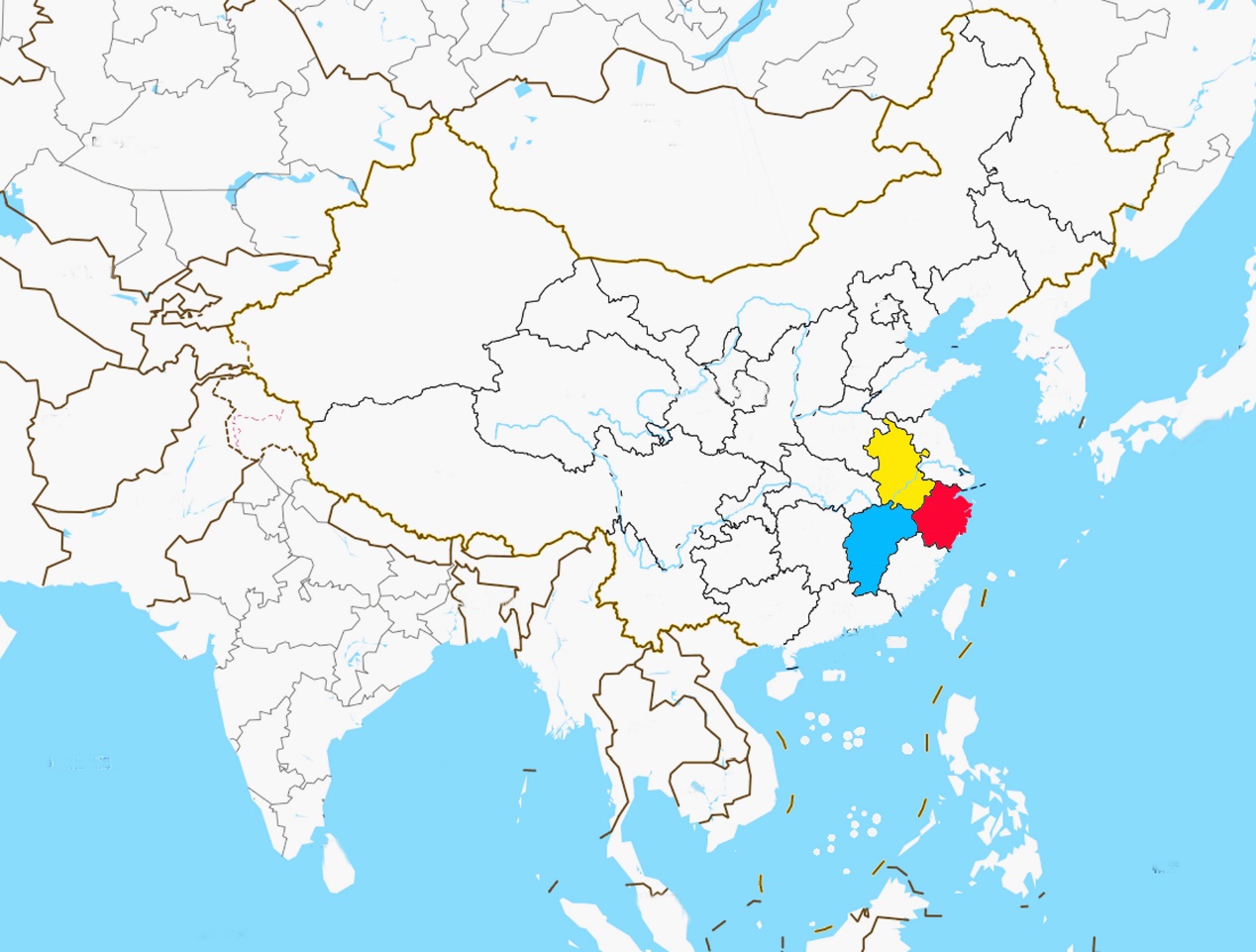


Figure S1 Geographical location of Zhejiang, Anhui and Jiangxi provinces in China. Red colour: Zhejiang province; Yellow: Anhui province; blue: Jiangxi province.


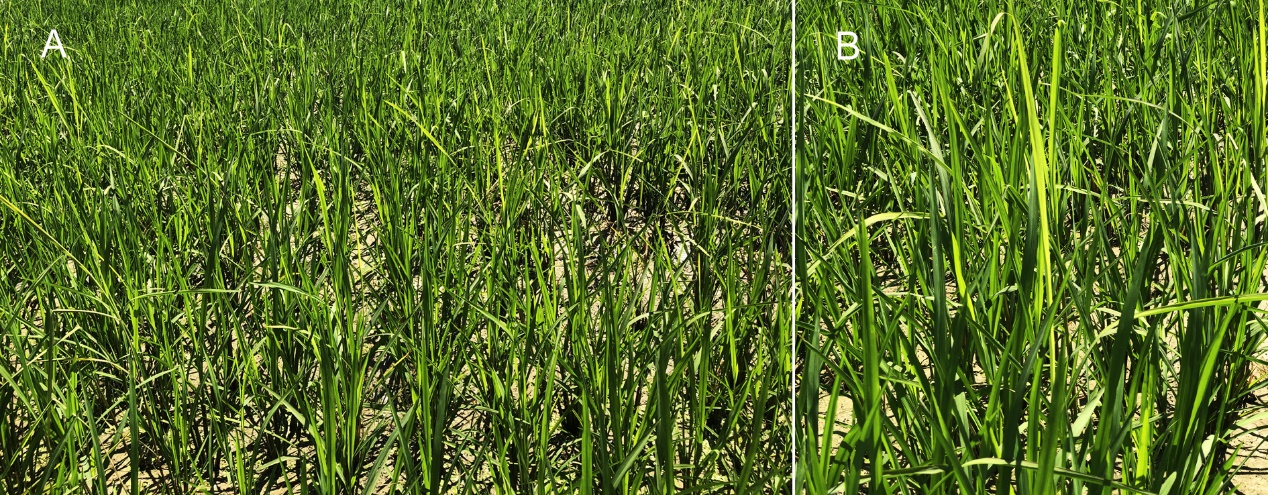


Figure S2 Typical symptoms of bakanae on rice seedlings in field. A. Abnormal elongation plants with chlorotic leaves. B. A partial magnification in Figure A.


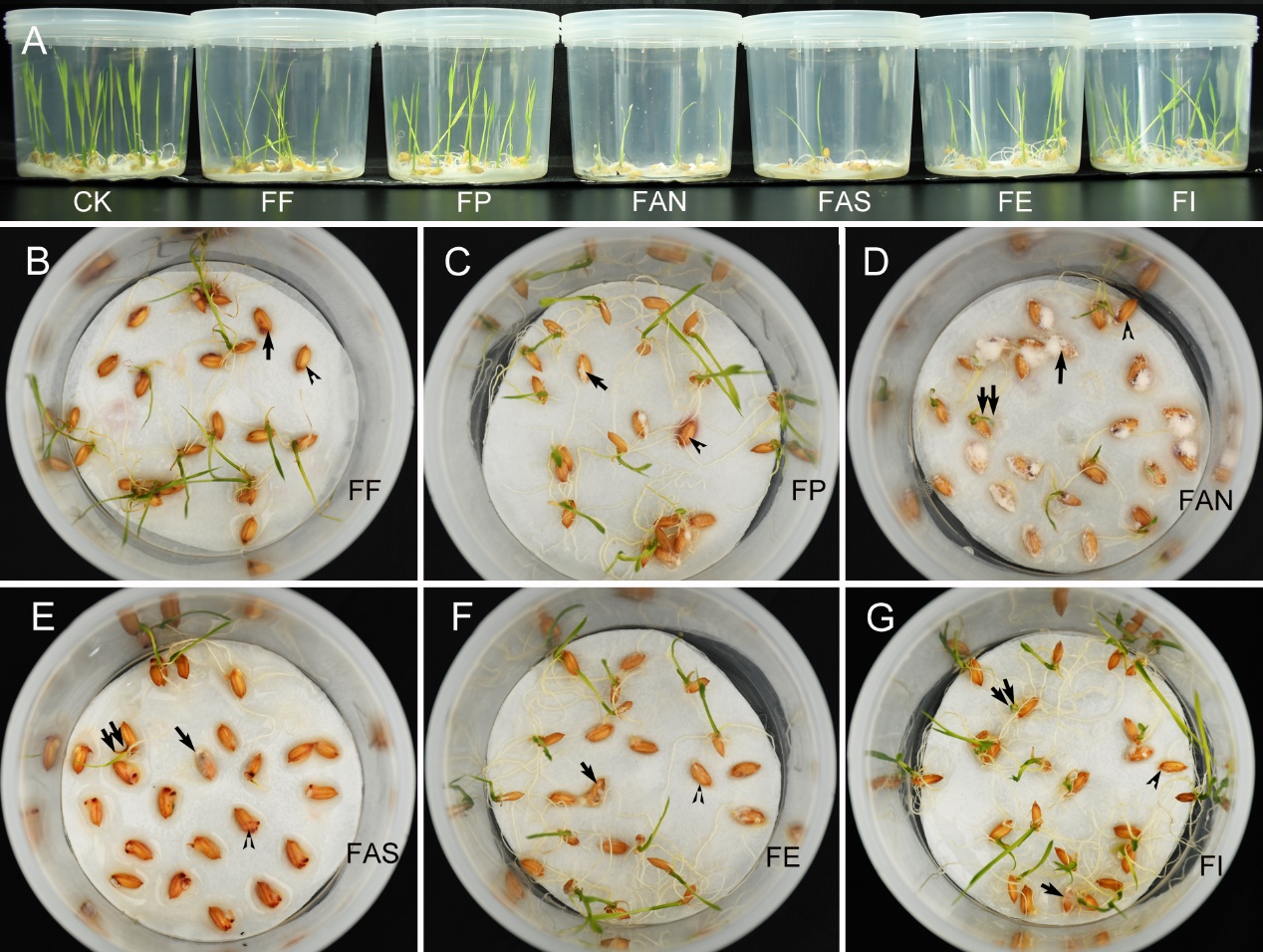


Figure S3 Inhibitory effect of different *Fusarium* spp. on rice seed germination 10 days after inoculation. CK: Control; FF: *F. fujikuroi* ZJ01; FAN: *F. andiyazi* ZJ08; FP: *F. proliferatum* ZJ05; FI: *F. incarnatum* ZJ11; FAS: *F. asiaticum* ZJ10; FE: *F. equiseti* ZJ09. Arrowheads: inbition of seed germination; arrows: seed rot; double arrows: bud rot.


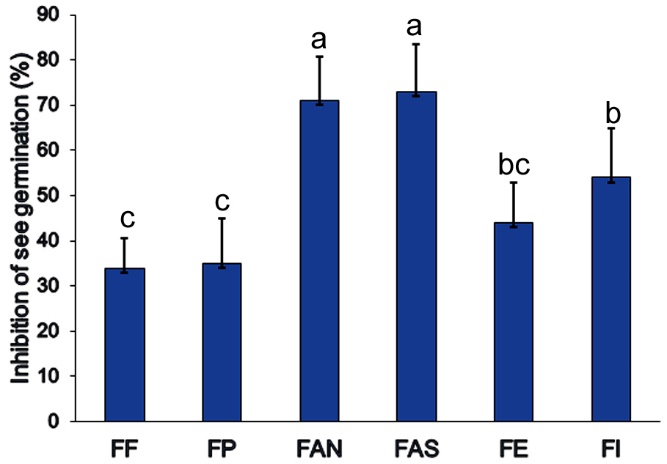


Figure S4. Percentage inhibition of seed germination after inoculation with isolates of *Fusarium fujikuroi* ZJ01, *F. proliferatum* ZJ05, *F. andiyazi* ZJ08*, F. asiaticum* ZJ10, *F. incarnatum* ZJ11 and *F. equiseti* ZJ09 for 10 days. The same letter(s) are not different significantly at P < 0.05 according to LSD.


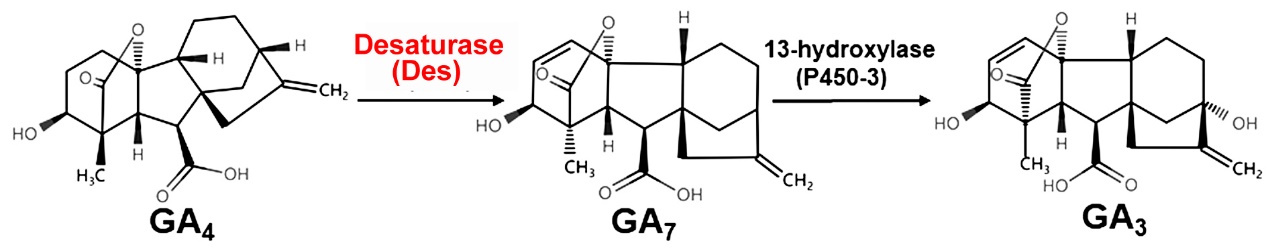


Figure S5 Substrate and product of desaturase marked in red in the last step of gibberellin biosynthesis pathways.


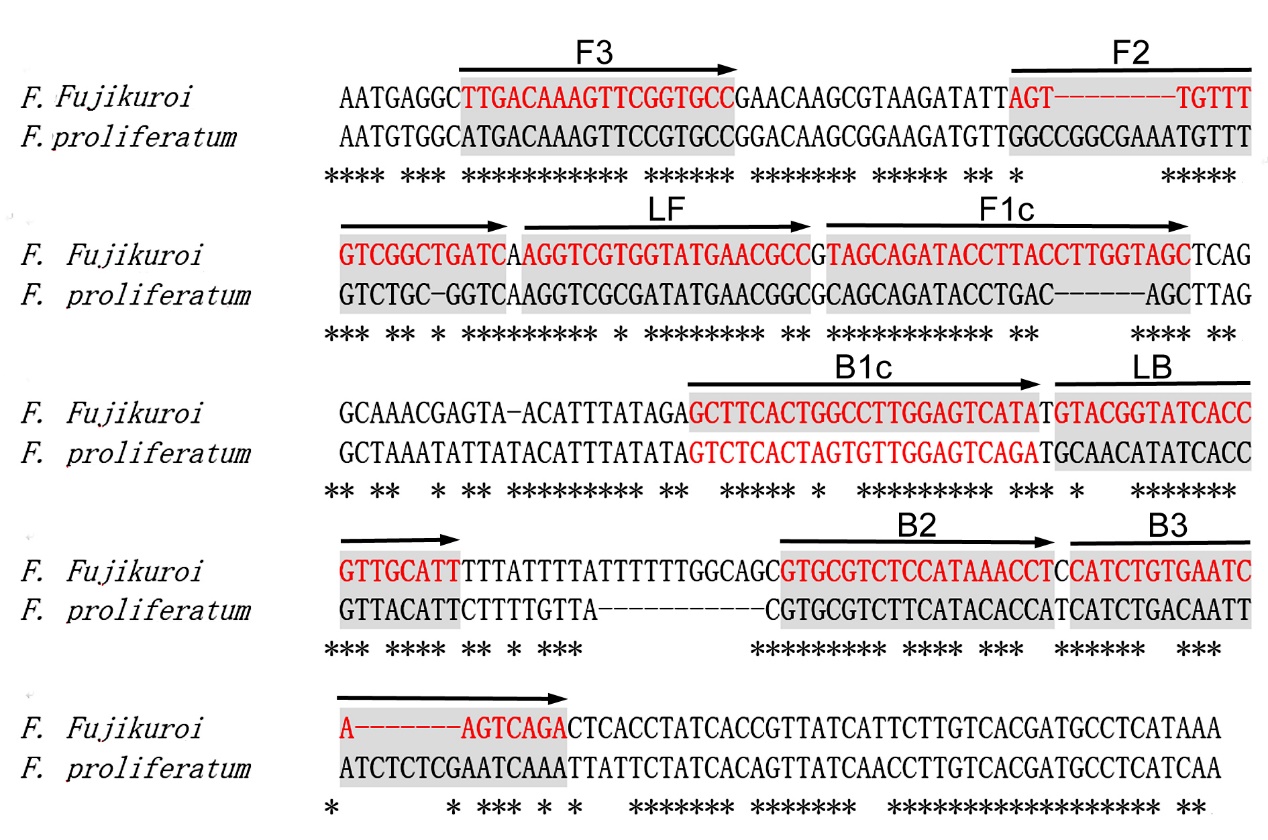


Figure S6 Partial sequences of the desaturase gene promoters of in *Fusarium fujikuroi* ZJ01and *F. proliferatum* ZJ05 and location of Loop-Mediated Isothermal Amplifcation (LAMP) primers. Arrows indicate LAMP primers (inner and outer primers) along with their position for detection of *F. fujikuroi*.


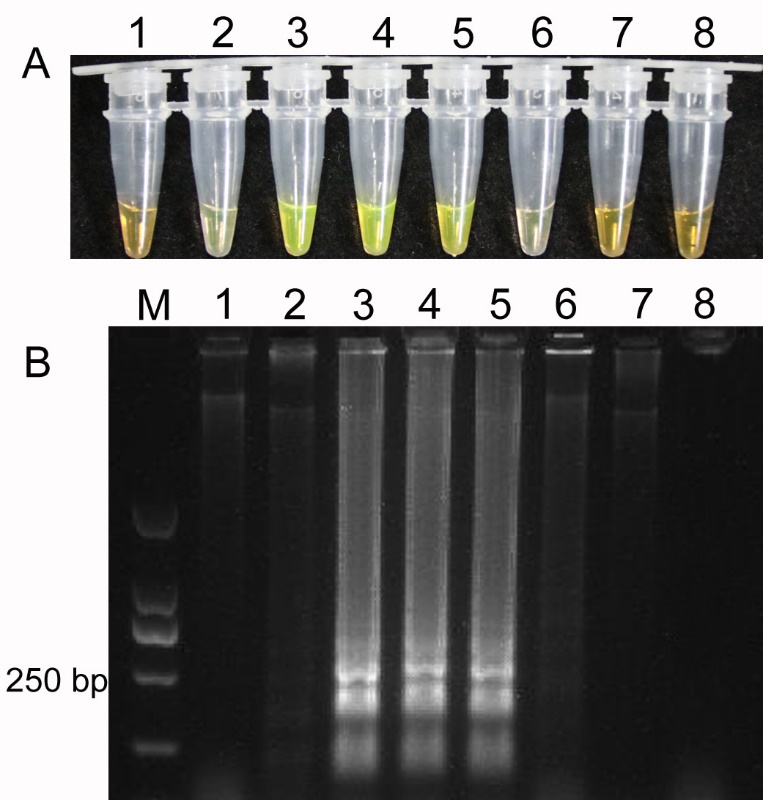


Figure S7 Detection of *F. fujikuroi* LAMP products under different temperature for 55 min. A. Visual inspection of LAMP reaction using SYBR Green I dye. The tubes from 1 to 8 are maintained at 50, 55, 60, 65, 67, 68, 69 and 70℃, respectively. B. Electrophoretogram of reaction products. Lanes 1-8 come from tubes 1-8. M, DNA marker.


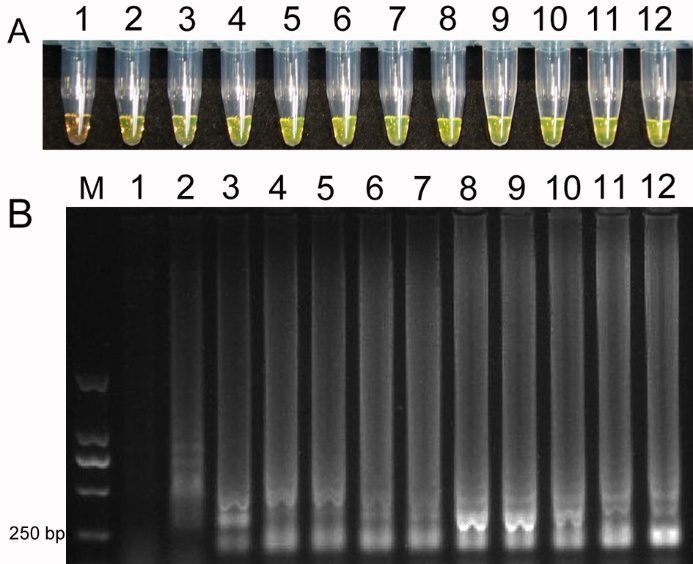


Figure S8 Detection of *F. fujikuroi* LAMP products at 65℃ for different time*.* A. Visual inspection. The tubes 2-12 are maintained for 20, 25, 30, 35, 40, 45, 50, 55, 60, 65, 70 and 75 min, respectively. Tuber 1: negative control. B. Electrophoretogram of LAMP products. Lanes 2-12, corresponding to tubes 2-12. Lane1: negative control. M: DNA marker.
