# Supplementary material for: Identification of Rice Seed-Derived Fusarium spp. and Development of LAMP Assay against Fusarium fujikuroi
Source: Pathogens. 2020 Dec 22;10(1):1. doi: 10.3390/pathogens10010001 (PMC7822049; doi:10.3390/pathogens10010001)
Supplement: Supplementary file 1 [file pathogens-10-00001-s001.zip › Supplemtary files/Supplementary Sequences/Supplementary Sequences.docx]

**Sequence**

1. Desaturase Gene from NBCI database

>AJ417493.1 Gibberella fujikuroi des gene for GA4 desaturase

AGAATCCAAATTCGATCCAGAATGCTTCAAAGTGACGTCCGAAGAGTTCGGATCAAGTCTACCAACTTCGGGGCGAGCACTATTCTTCCTAGGTTTCTTGGCAGTGTCCATCCTGAGTTAAACTCAATCACACATTTTCATTACTGAGCAACATCCAATTTTTCTCTTACTTGGATATCAATATCAGAAGAGAGAAGTGAAAGAGGAAGAGAAATCGTTGAGACATTCTAAGTCCGGGGACACGGTTGACCCCGAATCGTGGCTCTAACAAACTTCGCGAAGGGTCAAACAGCAAACCCTTGCAATTTCAAATGAACCAAACTCATGAGTCATCATTTCCGGATCGACTACCTCTTATGGGAAATAGTTTGAGGGTTCTGCATATGTGGCGTTGCTCATGTGCCGAGGTAAACATAGAAAATCTGACTGCCACTTCGGATAGTCCTGACATTGACTATTCATAAGCTTGACATTCATAGTTACTCCGACGATCTAGGCCGGCCCGTTGTACTAGTGAAGCTCGAAAAGAGGAGGTCTTGCGTTGCAATGGGAGGGTTACATACACATGCCATAGGTACACCCAGGGGTCTGAATTGTAACCACACGGCTGGTTATCAACAAAGCGCTTCTAGAACCACCCAAAGGACACGTCAATCTATCAGTCTGTCGGCAGACTACATTGTCACATTTTAGGCTTTCAAATAGCACATTAAAATGAGGCTTGACAAAGTTCGGTGCCGAACAAGCGTAAGATATTAGTTGTTTGTCGGCTGATCAAGGTCGTGGTATGAACGCCGTAGCAGATACCTTACCTTGGTAGCTCAGGCAAACGAGTAACATTTATAGAGCTTCACTGGCCTTGGAGTCATATGTACGGTATCACCGTTGCATTTTTATTTTATTTTTTGGCAGCGTGCGTCTCCATAAACCTCCATCTGTGAATCAAGTCAGACTCACCTATCACCGTTATCATTCTTGTCACGATGCCTCATAAAGATAATCTTCTTGAATCGCCAGTGGGCAAGAGTGTCACTGCTACTATAGCCTACCATAGCGGACCGGCTCTTCCAACCTCCCCGATCGCTGGTGTCACTACGCTCCAAGACTGCACTCAGCAGGCCGTAGCAGTGACTGATATCCGCCCTTCAGTCTCGTCCTTTACCCTAGATGGTAACGGCTTCCAGGTTGTCAAACATACATCGGCGGTAGGCTCTCCGCCGTATGATCACTCGTCGTGGACAGATCCAGTCGTTCGCAAGGAAGTGTATGACCCCGAAATCATTGAACTGGCAAAGTCTCTCACTGGAGCCAAGAAGGTCATGATTCTACTTGCTTCGTCTCGGAATGTTCCCTTCAAGGAGCCAGAGCTCGCCCCTCCTTATCCCATGCCTGGCAAATCAAGCAGCGGCAGCAAGGAAAGGGAAGCCATCCCAGCTAATGAGCTCCCTACTACAAGGGCAAAAGGTTTCCAAAAAGGCGAAGAGGAAGGCCCAGTACGAAAGCCTCATAAGGACTGGGGTCCATCCGGTGCGTGGAACACTCTCCGGAACTGGAGCCAAGAGCTCATTGATGAGGCTGGCGATATCATCAAGGCTGGCGATGAGGCTGCAAAGCTGCCAGGGGGCAGAGCAAAGAACTACCAAGGCAGACGATGGGCCCTGTATACTACCTGGCGTCCACTGAAAACTGTCAAGCGGGATCCCATGGCCTATGTAGACTACTGGACAGCTGATGAGGAAGATGGCGTGAGCTTCTGGCGTAACCCGCCAGGGGTGCATGGGACATTTGAGTCGGATGTACTACTTACCAAGGCTAATCCAAAGCATAAGTGGTACTGGATCAGTGACCAGACTCCGGATGAGGTTCTCCTCATGAAGATCATGGACACCGAGAGTGAGAAGGACGGGAGTGAAATAGCGGGAGGGGTTCACCACTGTTCATTTCATCTGCCGGGAACTGAGAAGGAGGAAGTGAGAGAGAGCATTGAGACCAAGTTCATTGCATTCTGGTAGTGGCTGTTTCTCTATATAATTATTATCTCCTAATTAACTCTATGAGATGACTGTTAGTTTCAATTTTCAATTACTATAAGGCGCGGATGCCTCATGCCATGCGATAATATTTTATTTCAGTTACATATTCCTCTAAATCTTCCTTTGTTCCAGGTGCTGACTTGCGAATCAGCCTCTTCCGTTTCCCCCCAACATATCGGCGAGCCTCACCCCACTTGAAACAGTATCGCTATTCCAAATGCGTTCCTCATCGTCATCATCAGTGACCGAAGCAGCTGTCACCGAGC

**Notes:** Red: coding region; green: primers (DesF:5’-GACACGGTTGACCCCGAATC-3’, DesR: 5’-GCAGTCTTGGAGCGTAGTGA-3’) for amplifying promoter region of desaturase; blue: LAMP marker (DesM_231_).

2. Partial sequences of desaturase promoter region amplified using primer pair DesF/DesR

F. proliferatum -CCGAATCGTGGCTATCA------CAAAGCGCCGCGGAGGGTTTAACAGCACACCCCTGC

F. Fujikuroi GCCGAATCGTGGCTCTAA------CAAA-CTTCGCGAAGGGTCAAACAGCAAACCCTTGC

F. oxysporum -CCGAATCGTGACTCCCAAGTCAACAAAACGTCGCGGAACGTCAAGCAACACACCCTTGC

********** ** * **** * **** * ** * ** ** **** ***

F. proliferatum AATCCCGAAAGAATCCAGTCTTATGAATCATCAATTACGAAT--CGACTTCCCTCTCATG

F. Fujikuroi AATTTCAAATGAA-CCAAACTCATGAGTCATCATTTCCGGAT--CGACTACC-TCTTATG

F. oxysporum AATATTCGGTGAAGCCATACTCACGGATCACCATTTCAGCTTAACCCCTCACTTTTTACG

*** *** *** ** * * *** ** ** * * * ** * * * * *

F. proliferatum GAAAATG---GTTTGAGGGTTCTGCATACGTGGCGTTGGTCATGTGCCGAGGTAAACATA

F. Fujikuroi GGAAATA---GTTTGAGGGTTCTGCATATGTGGCGTTGCTCATGTGCCGAGGTAAACATA

F. oxysporum GACGATAAAAGTTCGAGGCGTCTGGATACGTAGCATTGCTCATATGTGGAGGTGGACGTA

* ** *** **** **** *** ** ** *** **** ** ***** ** **

F. proliferatum GAATATTCAACTGCCACTTCGGGCATTCCCAGTATTGATTACGAATAAGCTTGGCACTCA

F. Fujikuroi GAAAATCTGACTGCCACTTCGGATAGTCCTGACATTGACTATTCATAAGCTTGACATTCA

F. oxysporum GAATATCCAACTGCCACTTTGGGCATTCCCGATTTCGAAGACTCACAGACTTGGCATTCA

*** ** ********** ** * *** * ** * * * **** ** ***

F. proliferatum TAATTACTCCGGCGATCTGGGCCGGCCCGTCGTAGTAGTGAACCTTGAAAAGAGGAGGTC

F. Fujikuroi TAGTTACTCCGACGATCTGGGCCGGCCCGTTGTACTAGTGAAGCTCGAAAAGAGGAGGTC

F. oxysporum TAAGTACTCCGGCGATCTAGACCGGCCCGTGGCCGCAAGGGAGCTTGAAAAGAGGAGGTC

** ******* ****** * ********* * * * * ** **************

F. proliferatum TTATGTCGCAATCTGTGAGGTTACATGCATGTGCCATGGGTATAGCCAGGGGTCGGAATT

F. Fujikuroi TTGCGTTGCAATGGGAG-GGTTACATACACATGCCATAGGTACACCCAGGGGTCTGAATT

F. oxysporum TTGCGTCACAATCTGGAAGGTTACATGCATATGCCATGGGTACACCCGGCGGTCGGAATT

** ** **** * ******** ** ****** **** * ** * **** *****

F. proliferatum GCAACCACACGGCTGGCTATCAATCAACCGCTTCCACAAACACATGAGGGACACGTCAA-

F. Fujikuroi GTAACCACACGGCTGGTTATCAACAAAGCGCTTCTAGAACCACCCAAAGGACACGTCAA-

F. oxysporum GCAGCCACACGCCTAGCTGTCAACCAAGCGCTTCTACAACCACCCAAGGGACACGTAAAA

* * ******* ** * * **** ** ****** * ** *** * ******** **

F. proliferatum TCTATCGG-TCTGTCGGCAGACAAAATTGTCACATTTTTGGTTCTCAATTCACAGACGCA

F. Fujikuroi TCTATCAG-TCTGTCGGCAGACTACATTGTCACATTTTAGGCTTTCAAATAGCACATTAA

F. oxysporum CCTCTCAGATCTGTCGTTAGACGATATCATCACATTAGATGTTTCTAAATGACAAGTGGA

** ** * ******* **** * ** ******* * * ** * ** *

F. proliferatum AATGTGGCATGACAAAGTTCCGTGCCGGACAAGCGGAAGATGTTGGCCGGCGAAATGT-T

F. Fujikuroi AATGAGGCTTGACAAAGTTCGGTGCCGAACAAGCGTAAGATATTAGT--------TGT-T

F. oxysporum AGTGAGACATGATAGAGTTTCGTGCCGAACAAGCGGAAGGTATTAGCCGGGAAAGTATGT

* ** * * *** * **** ****** ******* *** * ** * * * *

F. proliferatum TGTCTGC-GGTCAAGGTCGCGATATGAACGGCGCAGCAGATACC------TGACAGCTTA

F. Fujikuroi TGTCGGCTGATCAAGGTCGTGGTATGAACGCCGTAGCAGATACCTTACCTTGGTAGCTCA

F. oxysporum TGTCGGCTGATCAAGGTCGTGATGCGAACGGCGTAGTAGGTACCATGTATTAATAGCTTC

**** ** * ********* * * ***** ** ** ** **** * ****

F. proliferatum GGCTAAATATTATACATTTATATAGTCTCACTAGTGTTGGAGTCAGATGCAACATATCAC

F. Fujikuroi GGCAAACGAGTA-ACATTTATAGAGCTTCACTGGCCTTGGAGTCATATGTACGGTATCAC

F. oxysporum AGTAAAATATGATACGTTTA----------CTAGTCATCGACACATTTCTAAGATACGAG

* ** * * ** **** ** * * ** ** * * ** *

F. proliferatum CGTTACATTCTTTTGTTA-----------CGTGCGT-CTTCATACACCATCATCTGACAA

F. Fujikuroi CGTTGCATTTTTATTTTATTTTTTGGCAGCGTGCGT-CTCCATAAACCTCCATCTGTGAA

F. oxysporum GATTTCATTCTTTGGAAC---------AATTTGCCTTCTTCCTACACCGTTATCTGTCAA

** **** ** *** * ** * ** *** ***** **

F. proliferatum TTATCTCTCGAATCAAATTATTCTATCACAGTTATCAACCTTGTCACGATGCCTCATCAA

F. Fujikuroi TCA-------AGTCAGACTCACCTATCACCGTTATCATTCTTGTCACGATGCCTCATAAA

F. oxysporum TTTTCGCCCAAATCAAACTCGCTTATCACTTTTATCACCCTCGTCACGATGTCTCAGCAG

* * *** * * ****** ****** ** ********* **** *

F. proliferatum CATACTCCTCTTGAATCGCCAGTTGGCAAAAATGTCACTGCTACCATAGCCTACCACAGT

F. Fujikuroi GATAATCTTCTTGAATCGCCAGTGGGCAAGAGTGTCACTGCTACTATAGCCTACCATAGC

F. oxysporum GACACTCATCATGAATCGCCAGTTGGCAAGAATGTCACTGCTACTATCGCCTACCACAGT

* * ** ** ************ ***** * ************ ** ******** **

F. proliferatum GGACCGGCTCTTCCAACCTCCCCAATCGC-------

F. Fujikuroi GGACCGGCTCTTCCAACCTCCCCGATCGCTGGTGTC

F. oxysporum GGACCAGCTCTTCCAACCTCCCCAATCGCTGGTG--

***** ***************** *****

**Notes:** Grey region: LAMP marker (DesM_231_); red: coding region of desaturase
