# Supplementary material for: Identification of Rice Seed-Derived Fusarium spp. and Development of LAMP Assay against Fusarium fujikuroi
Source: Pathogens. 2020 Dec 22;10(1):1. doi: 10.3390/pathogens10010001 (PMC7822049; doi:10.3390/pathogens10010001)
Supplement: Supplementary file 1 [file pathogens-10-00001-s001.zip › Supplemtary files/Supplementary Table/Supplementary Table1,2,4,5.docx]

**Table S1** Rice varieties and origin in this study.

| No. | Name of varieties | Origin | Source of collection |
| --- | --- | --- | --- |
| 1 | Longlinagyouhuazhan | Quzhou city, Zhejiang province, China | Zhejiang seed Co. Ltd. |
| 2 | Wen 926 | Wenzhou city, Zhejiang province, China | Zhejiang seed Co. Ltd. |
| 3 | Jinzao 47 | Jinhua city, Zhejiang province, China | Zhejiang seed Co. Ltd. |
| 4 | Zhongjiazao17 | Wenzhou city, Zhejiang province, China | Zhejiang seed Co. Ltd. |
| 5 | Zhenuoyou 1 | Fuyang city, Zhejiang province, China | Zhejiang seed Co. Ltd. |
| 6 | Xiushui 14 | Jiaxing city, Zhejiang province, China | Zhejiang seed Co. Ltd. |
| 7 | Hanzhouzhongzao 39 | Hangzhou city, Zhejiang province, China | Zhejiang seed Co. Ltd. |
| 8 | Zhongzao 39 | Jinhua city, Zhejiang province, China | Zhejiang seed Co. Ltd. |
| 9 | Liangyou 619 | Changshan city, Zhejiang province, China | Zhejiang seed Co. Ltd. |
| 10 | Zhongzheyou1 | Jinhua city, Zhejiang province, China | Zhejiang seed Co. Ltd. |
| 11 | Shenliangyou 828 | Jinhua city, Zhejiang province, China | Zhejiang seed Co. Ltd. |
| 12 | Yongyou 1540 | Jinhua city, Zhejiang province, China | Zhejiang seed Co. Ltd. |
| 13 | Y-liangyou 17 | Jinhua city, Zhejiang province, China | Zhejiang seed Co. Ltd. |
| 14 | Zhongzu 3 | Wuhu city, Anhui province, China | Anhui seed Co. Ltd. |
| 15 | Zhongxian 310 | Wuhu city, Anhui province, China | Anhui seed Co. Ltd. |
| 16 | Zhongxian 402 | Wuhu city, Anhui province, China | Anhui seed Co. Ltd. |
| 17 | Zhongzao 39 | Wuhu city, Anhui province, China | Anhui seed Co. Ltd. |
| 18 | Zhendao18 | Wuhu city, Anhui province, China | Anhui seed Co. Ltd. |
| 19 | Wuyunjin 31 | Wuhu city, Anhui province, China | Anhui seed Co. Ltd. |
| 20 | Yijin 149 | Wuhu city, Anhui province, China | Anhui seed Co. Ltd. |
| 21 | Yihe 8 | Nanchang city, Jiangxi province, China | Jiangxi seed Co. Ltd. |
| 22 | Zhongbaiyouhuazhan | Nanchang city, Jiangxi province, China | Jiangxi seed Co. Ltd. |
| 23 | Ziyou 218 | Nanchang city, Jiangxi province, China | Jiangxi seed Co. Ltd. |
| 24 | Jiuliangyou 9815 | Nanchang city, Jiangxi province, China | Jiangxi seed Co. Ltd. |
| 25 | Yiyouhuazhan | Nanchang city, Jiangxi province, China | Jiangxi seed Co. Ltd. |

**Table S2** Strains of fungi used for testing the specificity in the loop mediates isothermal amplification (LAMP) and polymerase chain reaction (PCR) assays.

| **Species** | **Isolates** | **Host** | **Origins** |
| --- | --- | --- | --- |
| *F. Fujikuroi* | ZJ01 | Rice | Zhejiang |
| *F. proliferatum* | ZJ05 | Rice | Zhejiang |
| *F. equiseti* | ZJ09 | Rice | Zhejiang |
| *F. oxysporum* | ZJG10 | Rice | Zhejiang |
| *F. avenaceum* | ZJG | Abies beshanzuensis | Zhejiang |
| *F. asiaticum* | ZJ10 | Rice | Zhejiang |
| *F. graminearum* | ZJG12 | Rice | Zhejiang |
| *F. solani* | ZQ4R-2 | Rice | Zhejiang |
| *F. verticillioides* | ZJG15 | Rice | Zhejiang |
| *F. incarnatum* | ZJ11 | Rice | Zhejiang |
| *F. commune* | ZJG17 | Rice | Zhejiang |
| *F. Andiyazi* | ZJ07 | Rice | Zhejiang |
| *F. Boothii* | ZQ4R-4 | Abies beshanzuensis | Zhejiang |
| *Ustilago esculenta* | J26 | Zizania latifolia | Zhejiang |
| *Ustilago esculenta* | J6 | Zizania latifolia | Zhejiang |
| *Verticillium dahliae* | 086 | Cotton | Zhejiang |
| *Verticillium dahliae* | BP2 | Cotton | Zhejiang |
| Pyricularia oryzae | B157 | Rice | Zhejiang |
| *Ustilaginoidea oryzae* | ZJG06-4 | Rice | Zhejiang |
| *Sclerotinia sclerotiorum* | ZJG06-1 | Napus | Zhejiang |
| *Phytophthora capsici* | ZJG06-5 | Capsicum | Zhejiang |
| *Scopulariopsis gossypii* | ZJG06-2 | Cotton | Zhejiang |
| *Phytophthora nicotianae* | ZJG06-3 | *Tabacum* | Zhejiang |

**Table S4** Number and isolation frequency of *Fusarium* species from 25 rice varieties in three provinces

| No. | Varieties | *FF* | *FP* | *FE* | *FAS* | *FI* | *FAN* | *FC* |
| --- | --- | --- | --- | --- | --- | --- | --- | --- |
| Zhejiang province | | | | | | | | |
| 1 | Longlinagyouhuazhan | 25 | 0 | 0 | 2 | 3 | 0 | 0 |
| 2 | Wen 926 | 26 | 0 | 0 | 0 | 0 | 3 | 0 |
| 3 | Jinzao 47 | 31 | 3 | 0 | 0 | 0 | 0 | 0 |
| 4 | Zhongjiazao 17 | 10 | 7 | 0 | 0 | 3 | 0 | 0 |
| 5 | Zhenuoyou1 | 43 | 0 | 17 | 0 | 0 | 0 | 0 |
| 6 | Xiushui 14 | 8 | 0 | 0 | 0 | 0 | 0 | 7 |
| 7 | Hanzhouzhongzao 39 | 9 | 5 | 0 | 0 | 0 | 0 | 0 |
| 8 | Zhongzao39 | 7 | 0 | 0 | 0 | 0 | 1 | 0 |
| 9 | Liangyou619 | 37 | 1 | 0 | 0 | 0 | 0 | 0 |
| 10 | Zhongzheyou1 | 3 | 0 | 0 | 0 | 0 | 0 | 0 |
| 11 | Shenliangyou 828 | 8 | 0 | 0 | 0 | 0 | 0 | 0 |
| 12 | Yongyou1540 | 7 | 3 | 0 | 0 | 0 | 0 | 0 |
| 13 | Y-liangyou-17 | 7 | 5 | 0 | 0 | 0 | 0 | 0 |
| Anhui province | | | | | | | | |
| 14 | Zhongzu-3 | 5 | 0 | 1 | 0 | 0 | 0 | 0 |
| 15 | Zhongxian310 | 11 | 4 | 0 | 0 | 0 | 0 | 0 |
| 16 | Zhongxian402 | 13 | 0 | 0 | 0 | 0 | 0 | 0 |
| 17 | Zhongzao39 | 7 | 0 | 2 | 0 | 0 | 0 | 0 |
| 18 | Zhendao18 | 13 | 2 | 0 | 0 | 0 | 0 | 0 |
| 19 | Wuyunjin31 | 3 | 5 | 0 | 0 | 0 | 0 | 0 |
| 20 | Yijin149 | 43 | 0 | 5 | 0 | 0 | 0 | 0 |
| Jiangxi province | | | | | | | | |
| 21 | Yihe 8 | 2 | 0 | 0 | 0 | 0 | 0 | 0 |
| 22 | Zhongbaiyouhuazhan | 1 | 0 | 0 | 0 | 0 | 0 | 0 |
| 23 | Ziyou 218 | 5 | 0 | 0 | 0 | 0 | 0 | 0 |
| 24 | Jiuliangyou98 15 | 6 | 0 | 0 | 0 | 5 | 0 | 0 |
| 25 | Yiyouhuazhan | 7 | 0 | 0 | 0 | 0 | 0 | 0 |
| Statistics： | | | | | | | | |
| The total number of each species | | 337 | 35 | 25 | 2 | 11 | 4 | 7 |
| Percentage of each species | | 80.05% | 8.31% | 5.94% | 0.48% | 2.61% | 0.95% | 1.66% |

FF: *F. fujikuroi*; FP: *F. proliferatum*; FE: *F. equiseti:* FAS: *F. asiaticum:* FI: *F. incarnatum:* FAN: *F. andiyazi.* FC: *F. commune.*

**Table S5** the results of rice seed germination tests by inoculation with six Fusarium spp. Isolates.

| **Isolates** | **PI (%)** | **PSD (%)** | **PUS(%)** |
| --- | --- | --- | --- |
| FF | 33.8±6.5c | 27.5±6.5c | 6.3±5.4b |
| FP | 45.0±3.5b | 28.8±6.3c | 17.5±4.3a |
| FAN | 77.5±2.5a | 68.8±8.5a | 8.8±5.4b |
| FAS | 73.8±10.8a | 62.5±8.7a | 8.8±6.5b |
| FE | 53.8±11.4b | 37.5±9.6c | 16.3±6.5a |
| FI | 52.5±5.6b | 50.0±4.1b | 2.5±2.5b |
| CK | 100±0.0 | - | - |

PI：percentage inhibition (PI) of seed germination；percentage of sprouts decay (PSD)=[ number of sprouts decay/ total number of seeds] × 100%; percentage of ungerminated seeds (PUS) =[number of ungerminated seeds/ total number of seeds] × 100%. FF: *F. fujikuroi* ZJ01; FP: *F. proliferatum* ZJ05; FAN: *F. andiyazi* ZJ08, FAS: *F. asiaticum* ZJ10; FE: *F. equiseti* ZJ09; FI: *F. incarnatum* ZJ11.
